# High levels of endothelial ICAM-1 prohibit natalizumab mediated abrogation of CD4^+^ T cell arrest on the inflamed BBB under flow in vitro

**DOI:** 10.1186/s12974-023-02797-8

**Published:** 2023-05-23

**Authors:** Sasha Soldati, Alexander Bär, Mykhailo Vladymyrov, Dale Glavin, James L. McGrath, Fabien Gosselet, Hideaki Nishihara, Susan Goelz, Britta Engelhardt

**Affiliations:** 1 Theodor Kocher Institute, University of Bern, Freiestrasse 1, 3012 Bern, Switzerland; 2 Department of Biomedical Engineering, University of Rochester, Rochester, NY USA; 3 Blood-Brain Barrier Laboratory, University of Artois, Lens, France; 4 Biogen, Cambridge, MA USA; 5 Present Address: Department of Neurotherapeutics, Yamaguchi University, Yamaguchi, Japan

**Keywords:** Blood–brain barrier, Brain microvascular endothelial cells, Central nervous system, Extended interval dosing, Intercellular adhesion molecule-1, Multiple sclerosis, Natalizumab, Progressive multifocal leukoencephalopathy, Standard interval dosing, Vascular cell-adhesion molecule-1

## Abstract

**Introduction:**

The humanized anti-α4 integrin blocking antibody natalizumab (NTZ) is an effective treatment for relapsing–remitting multiple sclerosis (RRMS) that is associated with the risk of progressive multifocal leukoencephalopathy (PML). While extended interval dosing (EID) of NTZ reduces the risk for PML, the minimal dose of NTZ required to maintain its therapeutic efficacy remains unknown.

**Objective:**

Here we aimed to identify the minimal NTZ concentration required to inhibit the arrest of human effector/memory CD4^+^ T cell subsets or of PBMCs to the blood–brain barrier (BBB) under physiological flow in vitro*.*

**Results:**

Making use of three different human in vitro BBB models and in vitro live-cell imaging we observed that NTZ mediated inhibition of α4-integrins failed to abrogate T cell arrest to the inflamed BBB under physiological flow. Complete inhibition of shear resistant T cell arrest required additional inhibition of β2-integrins, which correlated with a strong upregulation of endothelial intercellular adhesion molecule (ICAM)-1 on the respective BBB models investigated. Indeed, NTZ mediated inhibition of shear resistant T cell arrest to combinations of immobilized recombinant vascular cell adhesion molecule (VCAM)-1 and ICAM-1 was abrogated in the presence of tenfold higher molar concentrations of ICAM-1 over VCAM-1. Also, monovalent NTZ was less potent than bivalent NTZ in inhibiting T cell arrest to VCAM-1 under physiological flow. In accordance with our previous observations ICAM-1 but not VCAM-1 mediated T cell crawling against the direction of flow.

**Conclusion:**

Taken together, our in vitro observations show that high levels of endothelial ICAM-1 abrogate NTZ mediated inhibition of T cell interaction with the BBB. EID of NTZ in MS patients may thus require consideration of the inflammatory status of the BBB as high levels of ICAM-1 may provide an alternative molecular cue allowing for pathogenic T cell entry into the CNS in the presence of NTZ.

**Supplementary Information:**

The online version contains supplementary material available at 10.1186/s12974-023-02797-8.

## Introduction

Multiple sclerosis (MS) is a chronic inflammatory disease of the central nervous system (CNS) affecting more than 2.8 million people worldwide [1, 2]. The major pathological hallmarks of MS are blood–brain barrier (BBB) breakdown, immune cell infiltration, demyelination, axonal injury, and neuronal loss [3, 4].

The most used animal model for MS, experimental autoimmune encephalomyelitis (EAE), has provided a better understanding of the patho-mechanisms underlying the acute phase of MS. EAE is initiated by neuroantigen-specific CD4^+^ T cells in susceptible animal strains [5]. CD4^+^ Th1, Th17 and Th1* cells (also referred to as Th17.1, Th1/Th17, exTh17 or Th1-like Th17 cells [6–9]) can transfer EAE and have been linked to disease onset, progression, and relapse rates also in MS [6, 10, 11]. While Th1 and Th17 cells are identified by the expression of the transcription factors T-bet and RORγt [8] as well as secretion of their signature cytokines interferon (IFN)-γ and interleukin (IL)-17, respectively, Th1* cells co-express T-bet and RORγt and secrete IFNγ and IL-17 [7, 12].

EAE models have also contributed to the development of multiple disease-modifying treatments, all targeting primarily the immune system [13]. Using an EAE model it was found that T cell interaction with the inflamed BBB requires α4-integrins [14]. These findings were translated into the clinic by the development of the α4-integrin blocking humanized monoclonal IgG4 antibody natalizumab (NTZ) [15, 16]. Therapeutic inhibition of α4-integrin mediated immune-cell trafficking across the BBB by NTZ has proven beneficial for the treatment of RRMS [17] and efficiently reduces relapse rates and disability progression as well as occurrence of new enlarging T2 lesions [18, 19].

Unfortunately, NTZ is associated with the risk of progressive multifocal leukoencephalopathy (PML), caused by the reactivation of the JC virus (JCV) resulting in strong focal demyelination within the CNS [20, 21]. It has thus been speculated that NTZ may also inhibit CNS entry of immune cells required to perform routine CNS immune surveillance.

In this context, it is relevant that a recent retrospective cohort study making use of data available from the Tysabri® Outreach: United Commitment to Health (TOUCH) Prescribing Program safety database, showed that extended-interval dosing (EID) of NTZ to 6 week intervals is associated with a significantly lower risk of developing PML compared to the standard interval dosing (SID), where MS patients receive an intravenous injection of 300 mg every 4 weeks. Furthermore, a model-based simulation [22] and several real-world studies suggested that EID of NTZ does not diminish its effectiveness on clinical and radiological outcomes when compared to SID [23–31], although variable definitions of EID were used (from 5 to 8 weeks intervals). A prospective randomized Phase3b clinical trial (NOVA) confirmed that most patients who are stable on 4-weekly SID dosing can switch to 6-weekly NTZ dosing with no loss of clinically detectable efficacy [31]. In an exploratory, dose- and frequency-blinded, prospective, randomized, dose-ranging study in RRMS (REFINE), EID of 12 weeks was, however, associated with increased MRI and relapse activity in patients, suggesting that an interval dose extension over 12 weeks becomes ineffective [32]. Furthermore, 4 cases of PML in patients with EID of NTZ were observed in the Italian PML cohort [33].

**Fig. 1:**
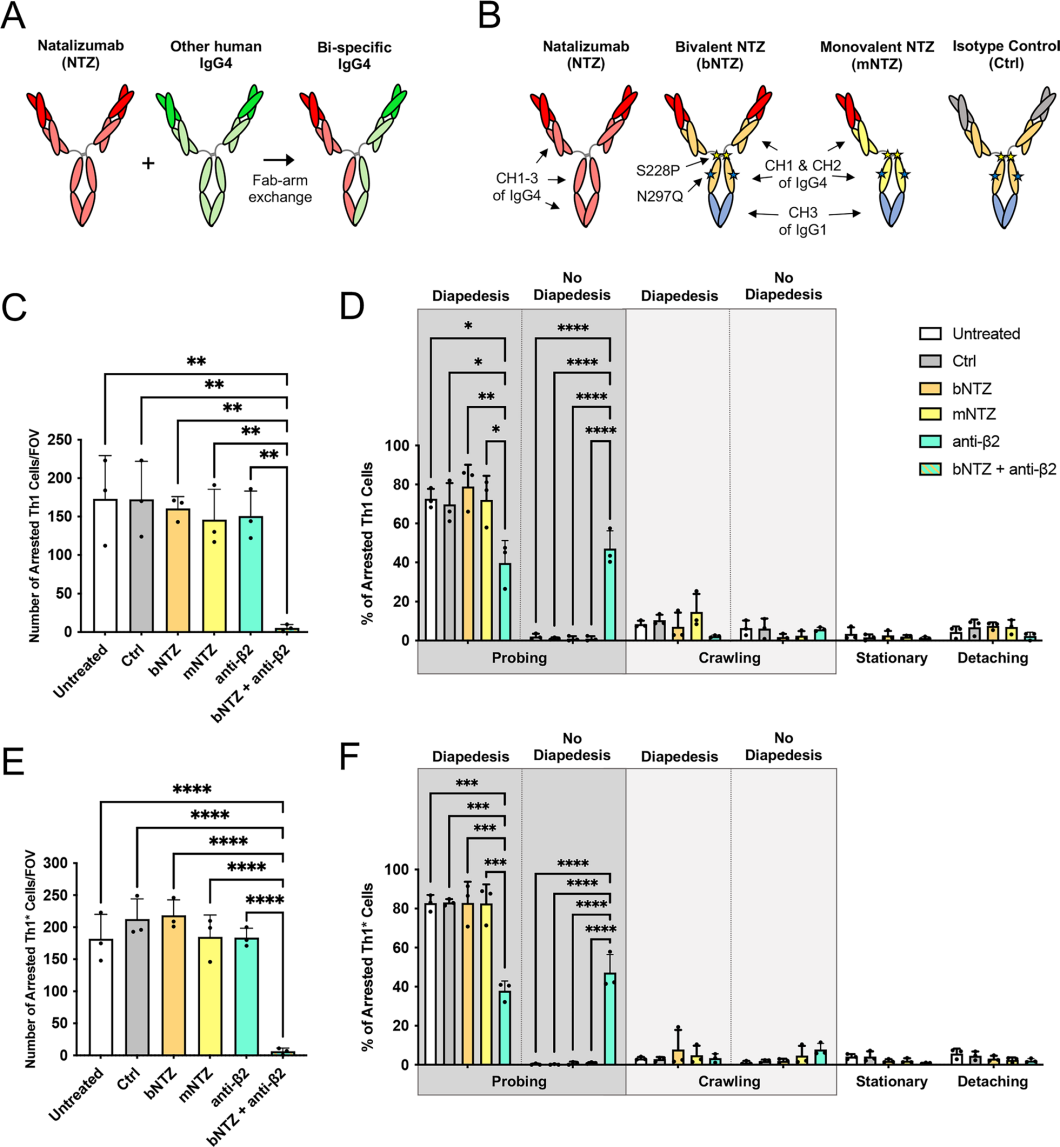
Inhibition of α4- and β2-integrins is required to inhibit T cell arrest on cytokine stimulated brain-like endothelial cells under flow. **A** Schematic representation of the Fab arm exchange between natalizumab (NTZ) and another human IgG4 antibody, resulting in a bi-specific IgG4 antibody with only one Fab arm specific for α4-integrins. **B** Schematic representation of the different NTZ constructs used in the present study. Natalizumab (NTZ) is the humanized anti-α4-integrin IgG4 antibody used in the clinic. The bivalent and monovalent NTZ constructs (bNTZ, 150 kDa; mNTZ, 100 kDa) contain the variable domains of NTZ but have instead a fixed hinge region and a hybrid Fc region formed by an aglycosyl IgG4P CH2 domain and an IgG1 CH3 domain. Point mutations (different from NTZ) within the hinge and CH1 domain are indicated by a yellow and blue star, respectively. Number of arrested human CD4^+^ Th1 (**C**) and Th1* (**E**) cells per field of view (FOV) on 16–24 h pro-inflammatory cytokine (76 IU/mL TNFα + 20 IU/mL IFNγ) stimulated brain-like endothelial cells (BLEC) under flow conditions. Th1 and Th1* cells were pre-incubated with equimolar concentrations of the different NTZ constructs (30 μg/mL bNTZ or 20 μg/mL mNTZ,) and/or with 1 μg/mL of a function-blocking anti-β2-integrin antibody. An isotype control antibody was used as internal control (30 μg/mL Ctrl). Quantification of the post-arrest dynamic behavior of human Th1 (**D**) and Th1* cells (**F**) on 16–24 h pro-inflammatory cytokine (76 IU/mL TNFα + 20 IU/mL IFNγ) stimulated BLEC under physiological flow conditions during 30 min of recording. Behavioral categories were subdivided in probing, crawling, stationary (not polarized), and detached and are shown as fraction of all shear-resistant arrested T cells (100%). Within the categories probing and crawling we additionally distinguished the categories with and without subsequent diapedesis. Post-arrest behavior of Th1 and Th1* cells treated with combined bNTZ and anti-β2-integrin antibody could not be analyzed due to the low numbers of arrested T cells. **C–F** Each figure shows the mean ± SEM of 3 independent experiments. Statistical analysis: one-way ANOVA followed by Tukey’s multiple-comparison test (p < 0.05 = *, p < 0.01 = **, p < 0.001 = ***, p < 0.0001 = ****)

Taken together, these observations suggests that lower serum concentrations of NTZ may still suffice to maintain its therapeutic efficacy, but at the same time reduce the risk of PML by allowing for a low level of T cell mediated CNS immune surveillance. It is thus mandatory to understand the lowest circulating concentration of NTZ required to maintain its therapeutic efficacy. In this context, it is also relevant to consider that NTZ shares with all IgG4 antibodies the unique ability to undergo “Fab-arm exchange”. As IgG4 immunoglobulins lack covalent links between their two heavy chains [34] they can swap a heavy chain-light chain pair with a heavy chain-light chain pair from another IgG4 leading to functionally bi-specific antibodies [35]. NTZ undergoes Fab-arm exchange with other circulating endogenous IgG4 antibodies in MS patients and “Fab-arm exchanged”-NTZ is found as the main circulating form already 4 weeks after injection [36]. Importantly, “Fab-arm exchanged”-NTZ thus functions in a monovalent form as with one paratope it still binds α4-integrins but the other paratope will bind to an unknown antigen **(**Fig. 1A**)** [36–39].

The present study therefore aimed to define the precise dose dependent effect of both bivalent and monovalent NTZ in inhibiting α4-integrin mediated interaction of different human CD4^+^ T cell subsets with purified VCAM-1 and with three different in vitro models of the human BBB under inflammatory conditions. We anticipated that defining the minimal and maximal efficacy of NTZ mediated inhibition of T cell interaction with VCAM-1 in the cellular context of the BBB will allow for optimized EID of NTZ tailored specifically to the individual patient by allowing to ensure the minimal serum concentrations required for therapeutic efficacy to reduce the risk of PML.

## Material and methods

### PBMCs isolation and T cell culture

PBMCs were isolated from buffy coats of healthy donors by Ficoll-Paque™ Plus (Cytiva) density gradient and were frozen and stored in a liquid nitrogen tank until use. Buffy coats were purchased from the Swiss Red Cross (Interregionale Blutspende SRK, Bern, Switzerland; project number P_172). Different human effector/memory CD4^+^ T helper cells (Th1, Th2, Th1*, and Th17) were isolated, sorted and expanded as described before [40–46]. Briefly, human CD4^+^ T cells were isolated by employing a CD4^+^ T cells isolation kit (Miltenyi Biotec kit) following provider’s instructions. Subsequently, effector/memory CD4^+^ T cells were sorted by Fluorescence-Activated Cell Sorting (FACS) into different Th subsets according to their specific surface expression of chemokine receptors (CCR6^−^CXCR3^+^CCR4^−^ for Th1; CCR6^+^CXCR3^+^CCR4^−^ for Th1*; CCR6^−^CXCR3^−^CCR4^+^ for Th2; CCR6^+^CXCR3^−^CCR4^+^ for Th17). The employed FACS antibodies can be found in Additional file 7: Table S1. Th cells were subsequently expanded for 20 days with periodic restimulation with 1 μg/mL phytohemagglutinin, 500 IU/mL recombinant human interleukin 2 (IL-2) and irradiated allogeneic peripheral blood mononucleated cells (PBMCs) in culture medium (RPMI-1640 (Gibco), 10% (v/v) heat inactivated fetal bovine serum (FBS, Hyclone), 2 mM L-Glutamine (Gibco), 1% (v/v) MEM Non-Essential Amino Acids Solution (Gibco), 1 mM Sodium Pyruvate (Gibco), and 0.05 mM β-Mercaptoethanol (Grogg Chemie AG), 10 U/mL Penicillin–Streptomycin (Gibco), 100 μg/mL Kanamycin Sulfate (Gibco)). Lastly, Th cells were frozen and stored in a liquid nitrogen tank until use.

One day prior experiment, Th cells were thawed and cultured overnight in culture medium at 37 °C (5% CO_2_). Th cells were then labelled or not with 1 μM CellTracker™ Green (CMFDA Dye, Life technologies) for 30 min at 37 °C (5% CO_2_). Dead cells were removed by Ficoll-Paque™ Plus (Cytiva) density gradient (780 × g, 20 min, 20 °C). Living cells were resuspended in migration assay medium (MAM) (DMEM w/o phenol red (Gibco), 5% (v/v) heat inactivated FBS (Hyclone), 4 mM L-Glutamine (Gibco), 25 mM HEPES (Gibco)) to 1 × 10^6^ cells/mL prior experiment.

### In vitro BBB models

#### Brain-like endothelial cells

Brain-like endothelial cells (BLEC) were used as a human in vitro model of the BBB as previously described [44–47]. The protocol for the handling of human tissues and cells was authorized by the French Ministry of Higher Education and Research (CODECOH Number DC2011-1321) and all patients gave their approval. In brief, CD34^+^ stem cells were isolated from human umbilical cord blood and differentiated to endothelial cells in ECM basal medium (ScienCell) umented with 20% (v/v) heat inactivated FBS (Life Technologies) and 50 ng/mL of VEGF165 (PeproTech Inc.). To induce BBB-like characteristics CD34^+^ endothelial cells were co-cultured with bovine pericytes or bovine pericyte conditioned medium. For immunocytochemistry and flow cytometry analysis, CD34^+^ endothelial cells were cultured to confluency on Matrigel™ coated filter inserts (PC membrane, pore size 0.4 μm; Costar, 3401) in co-culture with bovine pericytes for 6 days. For in vitro live-cell imaging, CD34^+^ endothelial cells were cultured to confluency on Matrigel™ coated nanoporous silicon nitride (NPN) membranes of μSiM-CVB flow chambers in co-culture with bovine pericyte conditioned medium for 6 days.

#### Human brain microvascular endothelial cells

Human brain microvascular endothelial cells (HBMEC) were kindly provided by Prof. Nicholas Schwab (University of Münster, Germany) and were cultured in speed-coating solution treated (PELOBiotech) T25 flasks using Cellovations® Microvascular Endothelial Cell Growth Medium Kit classic (PELOBiotech) following manufacturer’s instruction. For flow cytometry analysis, HBMEC were cultured to confluency on speed-coating solution coated T25 flasks for 2 days. For immunofluorescence stainings, HBMEC were cultured to confluency on speed coating solution coated filter inserts (PC membrane, pore size 0.4 μm; Costar, 3413) for 4 days. For in vitro live-cell imaging, HBMEC were cultured to confluency on speed coating solution coated µ-Dishes (35 mm, low, iBidi) for 2 days.

#### Extended endothelial culture method—brain microvascular endothelial cell-like cells

Brain microvascular endothelial cells (BMEC)-like cells were employed as a human in vitro model of the BBB. In brief, the previously published Extended Endothelial cell Culture Method (EECM) was used to differentiate human induced pluripotent stem cells (hiPSCs) to BMEC-like cells exactly as described [48–50]. hiPSCs were established from erythroblasts in the laboratory of Renaud DuPasquier (University of Lausanne, Switzerland) [51] and have previously been described [50, 52]. In this study we used hiPSCS from one healthy control (HC) (cell line ID: LNISi002-B) and one relapsing–remitting MS (RRMS) patient (cell line ID: LNISi007-B). For flow cytometry analysis, EECM-BMEC-like cells were cultured to confluency on collagen IV-coated 6-well plates (Costar) for 2 days. For immunofluorescence staining, EECM-BMEC-like cells were cultured to confluency on collagen IV and fibronectin coated filter inserts (PC membrane, pore size 0.4 μm; Costar, 3413) for 4 days. For in vitro live-cell imaging, EECM-BMEC-like cells were cultured to confluency on collagen IV and fibronectin coated µ-Dishes (35 mm, low, iBidi) for 2 days.

### Cell surface adhesion molecule expression and cell bound NTZ analysis by flow cytometry

BLEC, HBMEC and EECM-BMEC-like cells were cultured to confluency as described above. BLEC, HBMEC and EECM-BMEC-like cells were stimulated or not with 76 IU/mL of recombinant human TNFα (R&D systems, 210TA) and 20 IU/mL recombinant human IFN-γ (R&D systems, 285IF) for 16 h at 37 °C (5% CO_2_). In addition, HBMEC were stimulated with 10 IU/mL of recombinant human TNFα alone. The cell surface molecule expression of VCAM-1 and ICAM-1 was analyzed by flowcytometry exactly as described before [44, 48–50]. In brief, cells were washed once with HBSS (Gibco) supplied with 25 nM HEPES and gently detached using Accutase (Innovative cell technology). Subsequently, cells were washed and resuspended in FACS buffer (DPBS (Gibco), 2.5% (v/v) heat inactivated FBS, 0.1% (w/v) Sodium Azide (Sigma-Aldrich)). 2 × 10^5^ cells per condition were transferred to a 96-well microtiter plate and incubated with the fluorochrome conjugated antibodies or respective isotype controls for 30 min at 4 °C. After incubation, cells were washed twice with FACS buffer and measured with an Attune NxT Flow Cytometer (Thermofisher Scientific, Switzerland). Data were analyzed using FlowJoTM 10 software (Tree Star, Ashland, OR, USA). Detailed information about the employed antibodies can be found in Additional file 7: Table S2.

The cell surface molecule expression of α4-, β1- and β7- integrins on the different CD4^+^ Th subsets was analyzed by flowcytometry exactly as described above. Detailed information about the employed antibodies can be found in Additional file 7: Table S3.

T cell bound NTZ constructs and T cell bound anti-β2-integrin antibody (TS1/18, Invitrogen) were analyzed by flow cytometry using Cy^TM^3-conjugated goat anti-human IgG (H + L) (Jackson ImmunoResearch) and AlexaFluor^TM^647-conjugated goat anti-mouse IgG (H + L) (Invitrogen) respectively (exact antibody concentrations are illustrated in the figure legend).

### Immunofluorescence staining for cell surface adhesion molecules

BLEC, HBMEC and EECM-BMEC-like cells were cultured to confluency on filter inserts as described above. BLEC, HBMEC and EECM-BMEC-like cells were stimulated with 76 IU/mL of recombinant human TNFα (R&D systems, 210TA) and 20 IU/mL recombinant human IFNγ (R&D systems, 285IF) for 16 h at 37 °C (5% CO_2_). Immunofluorescence stainings for VCAM-1 and ICAM-1 on BLEC, HBMEC and EECM-BMEC-like cells were performed exactly as described before [44, 48–50].

In brief, primary antibodies for ICAM-1 and VCAM-1 were added to live cells and incubated for 15 min at 37 °C (5% CO_2_). After washing, cells were fixed with 1% (w/v) paraformaldehyde and blocked with 5% (w/v) skimmed milk in PBS. BLEC were additionally incubated with a primary antibody for ZO-1 in 5% (w/v) skimmed milk with 0.2% Triton-X in PBS for 1 h at RT. After washing with DPBS, BLEC, HBMEC and EECM-BMEC-like cells were then incubated with fluorochrome-labelled secondary antibodies for 1 h at RT. Nuclei were stained with 1 μg/mL DAPI. After washing with PBS, filters inserts were mounted with Mowiol (Sigma-Aldrich) to a glass slide and imaged using a Nikon Eclipse E600 microscope connected to a Nikon Digital Camera DXM1200F with Nikon NIS-Elements BR3.10 software (Nikon, Egg, Switzerland). Detailed information about the employed antibodies can be found in Additional file 7: Table S4.

### T cell binding assays under static conditions

T cell binding assays to recombinant BBB cell adhesion molecules under static condition were performed as described before [53–56]. In brief, Teflon (PTFE) slides (Thermofisher Scientific) were directly coated with 10 μg/mL of either human recombinant VCAM-1 (Biolegend), human recombinant JAM-B (R&D system) or human recombinant fibronectin (R&D system) in DPBS for 1 h at 37 °C and blocked with 1.5% (v/v) bovine serum albumin (Sigma-Aldrich) in DPBS over night at 4 °C. Precoating with Protein A was omitted due to potential interactions with the Fc region of the different natalizumab (NTZ) constructs. Recombinant Delta/Notch-like EGF-related receptor (R&D system) was employed as negative control to test for unspecific T cell interactions.

CMFDA pre-labelled Th cells were incubated with the different NTZ constructs (Fig. 1B) provided by Biogen (Cambridge, MA, USA) for 30 min at 37 °C (5% CO_2_) (exact antibody concentrations are illustrated in the figure legend) and let adhere to the immobilized recombinant adhesion molecules for 30 min at RT with gentle shacking. Slides were then washed twice with PBS and fixed with 2.5% (v/v) glutaraldehyde in PBS for 2 h on ice. Adhered T cells were imaged using a Nikon Eclipse E600 microscope connected to a Nikon Digital Camera DXM1200F with Nikon NIS-Elements BR3.10 software (Nikon, Egg, Switzerland) and counted using Image J software (NIH, Bethesda, MD, USA).

### T cell binding assays under physiological flow conditions

For live-cell imaging of T cell interaction with recombinant BBB cell adhesion molecules under physiological flow condition, µ-Dishes (35 mm, low, iBidi) were coated with 0.01–50 μg/mL human recombinant VCAM-1 (Biolegend) and/or 0.01–50 μg/mL human recombinant ICAM-1 (R&D system) as described above (exact concentrations are illustrated in the figure legend).

CMFDA prelabelled Th cells were incubated with the different NTZ constructs and/or an anti- β2-integrin antibody (TS1/18, Invitrogen), and respective isotype control antibody for 30 min at 37 °C (8% CO_2_) prior imaging (exact antibody concentrations are illustrated in the figure legend). Th cells were subsequently perfused on top of the pre-coated dishes at a concentration of 10^6^/mL in MAM as previously described [54]. In brief, a parallel flow chamber connected to an automated syringe pump (Harvard Apparatus, Holliston, MA, USA) was mounted on the pre-coated dishes and placed on the heating stage of an inverted microscope. Th cells were then allowed to accumulate for 4 min at low shear stress (0.1 dyn/cm^2^) and their interaction with VCAM-1 and/or ICAM-1 was imaged under physiological shear stress (1.5 dyn/cm^2^) using an AxioObserver Z1 microscope (Carl Zeiss, Feldbach, Switzerland) connected to a digital camera (Carl Zeiss). Time-lapse videos were created by taking one image every 10 s over a 9 min period using the ZEN blue software (AxioVision, Carl Zeiss). Off-line video analysis allowing to define the number of adhered CMFDA pre-labelled Th cells and their behavior (e.g., mean crawling speed, mean crawling distance, and mean crawling Euclidian distance) was performed using Image J software (NIH, Bethesda, MD, USA) and Chemotaxis and Migration Tool (iBidi). Directionality of Th crawling on VCAM-1 and ICAM-1 under physiological shear stress was calculated as forward migration index towards the x-axis (xFMI), where xFMI is the straight x-axis distance (Dx) covered by the Th1* cell divided by the accumulated total distance (Dacc) of Th movement (xFMI = Dx/Dacc).

### T cell transmigration assays under physiological flow conditions

BLEC were grown to confluency in μSiM-CVB flow chambers as described above. HBMEC and EECM-BMEC-like cells were culture to confluency in µ-Dishes as illustrated before. BLEC and HBMEC were stimulated with 76 IU/mL of recombinant human TNFα (R&D systems, 210TA) and 20 IU/mL recombinant human IFNγ (R&D systems, 285IF) for 16–24 h at 37 °C (5% CO_2_). EECM-BMEC-like cells were stimulated with 7.6 IU/mL of recombinant human TNFα (R&D systems, 210TA) and 2 IU/mL recombinant human IFNγ (R&D systems, 285IF) in the presence of hiPSC derived smooth muscle-like cell’s (SMLCs) conditioned medium from the same donor for 16–24 h at 37 °C (5% CO_2_). hiPSC derived SMLCs and conditioned medium from SMLCs were obtained as previously described [48–50]. CMFDA prelabelled Th cells or PBMCs were incubated with the different NTZ constructs and/or an anti-β2-integrin antibody (TS1/18, Invitrogen), and respective isotype control antibody for 30 min at 37 °C (8% CO_2_) (exact antibody concentrations are illustrated in the figure legend). In our experiments using BLEC, Th1 and Th1* cells were preincubated with equimolar concentrations of bNTZ or mNTZ (30 or 20 μg/mL, respectively), based on the NTZ serum levels observed in RRMS patients at the time of NTZ re-dosing [57]. Th cells or PBMCs were subsequently perfused on top of BLEC, HBMEC and EECM-BMEC-like cells at a concentration of 10^6^/mL in MAM as previously described [46, 48]. In brief, flow was applied by connecting each flow chambers to an automatic syringe pump (Harvard Apparatus, Holliston, MA, USA) and mounted on the heating stage of an inverted microscope. Th cells or PBMCs were then allowed to accumulate for 4 min at low shear stress (0.1 dyn/cm^2^) and their interaction with BLEC, HBMEC and EECM-BMEC-like cells was imaged under physiological shear stress (1.5 dyn/cm^2^) using an AxioObserver Z1 microscope (Carl Zeiss, Feldbach, Switzerland) connected to a digital camera (AxioVision, Carl Zeiss). Time-lapse videos were created by taking one image every 5 s over a 30 min period for BLEC, or every 10 s over a 9 min period for HBMEC and EECM-BMEC-like cells using the ZEN blue software (Carl Zeiss). Off-line video analysis allowing to define the arrest of Th cells or PBMCs on BLEC, HBMEC and EECM-BMEC-like cells and the post-arrest behavior of Th cells on BLEC was categorized as probing, crawling, and diapedesis as described before [46, 54, 55] and analyzed with Image J software (NIH, Bethesda, MD, USA) Th cells crawling out of the FOV during image acquisition were not included in the behavioral analysis.

### Bioinformatics analysis workflow

Raw RNA-seq read data from Song et al. 2020 [58] (BioProject PRJNA596224, 12 samples) was downloaded from the NCBI Sequence Read Archive. The quality of the RNA-seq data was assessed using FastQC v.0.11.9 [59] and RSeQC v.4.0.0 [60]. The reads were mapped to the reference genome (Mus_musculus.GRCm39 and Homo_sapiens.GRCh38 for mouse and human samples respectively) using HiSat2 v.2.2.1 [61]. FeatureCounts v.2.0.1 [62] was used to count the number of reads overlapping with each gene as specified in the genome annotation (Mus_musculus.GRCm39.108 and Homo_sapiens.GRCh38.107). Using the total length of non-overlapping exons for each gene provided by FeatureCounts, raw read counts were converted to normalized transcripts per million (TPM) [63] with the ‘convertCounts’ function of the Differential Gene Expression (DGE) Analysis Utility Toolkit package in R version 4.2.1 [23, 64].

### Statistical analysis

Data are shown as the mean ± SD or SEM. Statistical significance between two groups was assessed by unpaired T-test, while comparison between multiple groups was assessed by one-way ANOVA followed by Tukey’s multiple-comparison test or by Dunn’s multiple-comparison test (Kruskal–Wallis’s test). Precise statistical analysis and p-values are indicated in the corresponding figures and figure legends (p < 0.05 = *, p < 0.01 = **, p < 0.001 = ***, p < 0.0001 = ****). Statistical analyses comprising calculation of degrees of freedom were done using GraphPad Prism 9 software (Graphpad software, La Jolla, CA, USA).

## Results

### Inhibition of both α4- and β2-integrins is required to inhibit T cell arrest to cytokine stimulated human BBB models in vitro

Our previous studies identified Th1 and Th1* cells when compared to Th17 and Th2 cells to preferentially migrate across human brain-like endothelial cells (BLEC) under static conditions [44]. To define the effect of NTZ in inhibiting CD4^+^ T cell arrest to and migration across the BBB under physiological flow, we therefore first studied the effect of bivalent NTZ (bNTZ) and monovalent NTZ (mNTZ) on the interaction of Th1 and Th1* cells with cytokine stimulated BLEC under physiological flow conditions by in vitro live-cell imaging (Fig. 1B). To our surprise neither bNTZ nor mNTZ significantly reduced the arrest of Th1 (Fig. 1C) or Th1* cells (Fig. 1E) on BLEC under flow. At the same time, antibody mediated inhibition of β2-integrins, mediating binding to endothelial ICAM-1 and ICAM-2, also failed to significantly reduce Th1 and Th1* cell arrest on BLEC under flow (Fig. 1C, E). Complete inhibition of the shear resistant arrest of the different Th subsets to BLEC could only be achieved by combined inhibition of α4- and β2-integrins (Fig. 1C, E; Additional file 2: Movie S1). After their shear resistant arrest, the majority of Th1 and Th1* cells (75.10 ± 5.09 and 83.05 ± 3.50%) probed the BLEC surface to find a site permissive for diapedesis (Fig. 1D, F). On the other hand, only 17.09 ± 7.23% of Th1 and 5.27 ± 3.51% of Th1* cells crawled on the BLEC surface prior to diapedesis. Furthermore, most of the shear resistant arrested Th1 and Th1* cells underwent diapedesis (respectively 81.13 ± 5.17 and 86.06 ± 3.54%) (Fig. 1D, F). While the different NTZ constructs did not significantly affect post-arrest behavior of Th1 (Fig. 1D) and Th1* cells (Fig. 1F), blocking β2-integrins significantly reduced diapedesis of Th1 and Th1* cells following probing and crawling, resulting in a significantly increased fraction of Th1 and Th1* cells probing the surface of BLEC (Fig. 1D, F). Post-arrest behavior of the few Th1 and Th1* cells able to arrest on BLEC despite inhibition of α4- and β2-integrins could not be analyzed due to the low number of events.

### Natalizumab mediated dose-dependent inhibition of T cell adhesion to VCAM-1 under static conditions

**Fig. 2:**
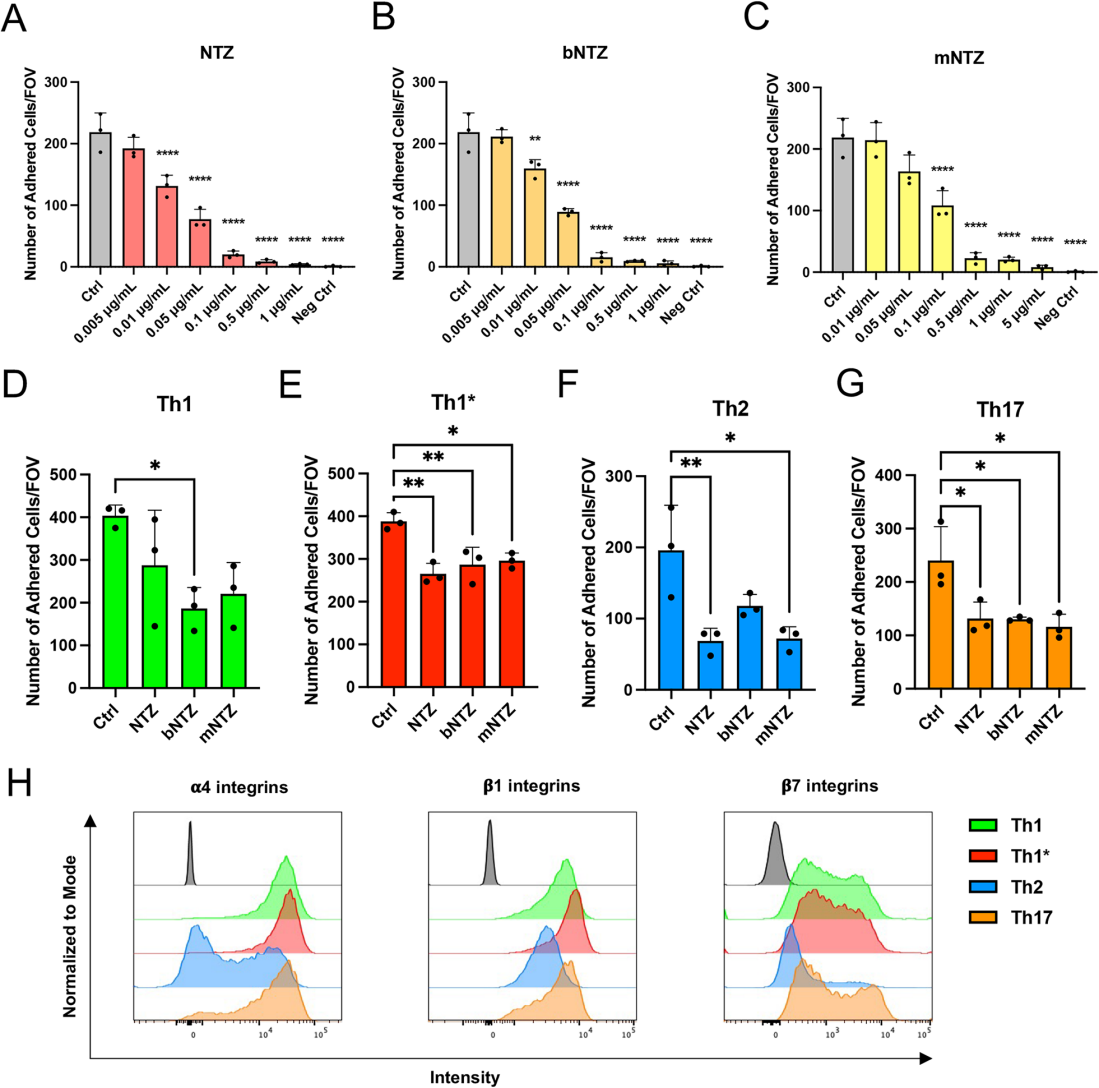
NTZ reduces T cell adhesion to immobilized recombinant VCAM-1 in a dose dependent manner. Number of human CD4^+^ Th1 cells adhering to immobilized recombinant VCAM-1 under static conditions. Th1 cells were pre-treated with titrated concentrations of natalizumab (NTZ) (**A**), bNTZ (**B**) and mNTZ (**C**) as indicated. An isotype control antibody was used as internal control (1 μg/mL Ctrl). Immobilized recombinant DNER was used as negative control (Neg Ctrl). Statistically significant differences are shown only for the comparison with the Ctrl condition. Number of adhered human Th1 (**D**), Th1* (**E**), Th2 (**F**) and Th17 cells (**G**) on immobilized recombinant VCAM-1 under static conditions. Th1* cells were treated with the minimal significant inhibitory concentration of NTZ (0.01 μg/mL), bNTZ (0.01 μg/mL) and mNTZ (0.1 μg/mL) found in the prior experiment (**A**–**C**). An isotype control antibody was used as internal control (0.01 or 0.1 μg/mL Ctrl). **A**–**G** Each figure shows the mean ± SEM of 3 independent experiments. Statistical analysis: one-way ANOVA followed by Tukey’s multiple-comparison test (p < 0.05 = *, p < 0.01 = **, p < 0.001 = ***, p < 0.0001 = ****). **H** Multicolor flow cytometry analysis for α4 -, β1- and β7-integrin cell-surface expression on human Th1 (green), Th1* (red), Th2 (blue) and Th17 cells (orange). Histogram plots are representative for 3 individual experiments

Since both, bNTZ and mNTZ failed to significantly reduce Th1 and Th1* cell arrest to BLEC under flow, we next compared the dose dependent inhibition of commercially available NTZ, bNTZ and mNTZ on Th1 cell adhesion to immobilized recombinant VCAM-1 under static conditions. To this end, we first verified by flow cytometry that all NTZ constructs showed saturating α4-integrin binding on the T cell surface at 1 μg/mL (Additional file 1: Fig. S1A). Similar results were also obtained for the anti-β2-integrin antibody. As expected, we observed that all NTZ constructs abrogated Th1 cell adhesion to VCAM-1 in a dose dependent manner (Fig. 2A–C). The minimal significant inhibitory concentration of NTZ and bNTZ was 0.01 μg/mL (Fig. 2A, B), while that of mNTZ was with 0.1 μg/mL tenfold higher (Fig. 2C), underscoring the more potent inhibitory activity of NTZ and bNTZ when compared to mNTZ. Accordingly, NTZ and bNTZ inhibited Th1 cell adhesion to VCAM-1 to more than 95% at a concentration of 0.5 μg/mL (Additional file 1: Fig. S1 B, C) compared to the tenfold higher concentration of 5 μg/mL required by mNTZ (Additional file 1: Fig. S1D).

Taken together, an almost tenfold higher concentration of mNTZ when compared to NTZ and bNTZ was required to exert a minimal significant inhibitory activity on α4-integrin mediated adhesion of CD4^+^ T cells to VCAM-1 underscoring the lower inhibitory efficacy of monovalent NTZ.

To understand if the minimal inhibitory concentrations of NTZ, bNTZ and mNTZ blocking Th1 cell adhesion to VCAM-1 would equally affect the adhesion of Th1*, Th2 and Th17 cells we next compared side-by-side the adhesion of these Th cell subsets to VCAM-1. In general, higher numbers of Th1 (Fig. 2D) and Th1* cells (Fig. 2E) adhered to VCAM-1 compared to Th2 (Fig. 2F) and Th17 cells (Fig. 2G**,** Additional file 1: Fig. S1E). The minimal inhibitory concentrations of NTZ, bNTZ and mNTZ defined to reduce Th1 cell binding to VCAM-1 (Fig. 1A–C) in general also reduced the adhesion of Th1*, Th17 and Th2 cells to VCAM-1, although a significant inhibition of adhesion to VCAM-1 by the minimal inhibitory concentrations with all NTZ constructs was only observed for Th1* and Th17 cells (Fig. 2D–G). Adhesion of Th1 cells to VCAM-1 was only significantly reduced by the minimal inhibitory concentration of bNTZ. Overall, these observations demonstrate that NTZ efficiently reduces the adhesion of different Th subpopulations to VCAM-1 with potentially different specific efficacy among subtypes.

To explore if the latter is due to different cell-surface levels of α4β1- and α4β7-integrins, we next analyzed cell surface integrin expression of Th1, Th1*, Th2 and Th17 cells by flowcytometry. Th1 and Th1* cells showed a high and homogenous surface expression of α4-integrins (Fig. 2H, Additional file 1: Fig. S1F-I), while Th2 and Th17 cells divided in two subsets expressing high and low levels of α4-integrins, with Th2 cells showing the lowest α4-integrins cell-surface expression (Fig. 2H**,** Additional file 1: Fig. S1G, I). Similarly, the surface expression of β1-integrins was the highest on Th1* cells over Th1 and Th17 cells and the lowest on Th2 cells (Fig. 2H, Additional file 1: Fig. S1G, I). While the majority of Th2 cells showed low β7-integrin cell surface levels (Additional file 1: Fig. S1G, I), Th1, Th1* and Th17 cells divided in two populations with high and low cell surface levels of β7-integrins (Fig. 2H, Additional file 1: Fig. S1G, H). The high cell surface levels of α4- and β1-integrins detected on Th1 and Th1* cells when compared to Th2 and Th17 cells correlate well with their higher binding capacity to VCAM-1 (Fig. 1D–G). Efficacy of the respective NTZ constructs in inhibiting the adhesion of CD4^+^ T cells to VCAM-1 may thus also depend on the cell surface levels of α4β1-integrins expressed on the respective Th subsets.

In addition, affinity of α4β1-integrin mediated binding to endothelial ligands other than VCAM-1 need to be considered for therapeutic efficacy of NTZ. Therefore, we tested adhesion of Th1 cells expressing high cell surface levels of α4- and β1-integrins to fibronectin [65, 66] and junctional adhesion molecule B (JAM-B) [67], which both have been shown to mediate T cell migration across the BBB [68, 69]. While Th1 cells readily adhered to fibronectin (Additional file 1: Fig. S2B), only few Th1 cells bound to JAM-B (Additional file 1: Fig. S2A). Interestingly, neither Th1 adhesion to fibronectin nor to JAM-B was reduced by 1 μg/mL NTZ (Additional file 1: Fig. S2A, B) suggesting that adhesion molecules other than α4-integrins dominate Th1 cell adhesion to these molecules.

### Combined inhibition of α4- and β2-integrins is required to inhibit T cell arrest to inflamed human brain microvascular endothelial cells

**Fig. 3:**
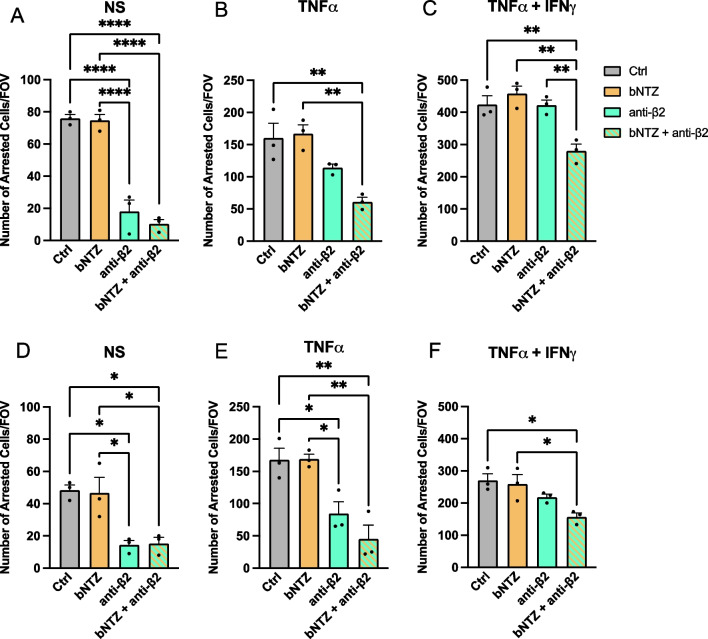
Inhibition of both α4- and β2-integrins is required to reduce T cell or PBMC arrest on human brain microvascular endothelial cells under flow. Number of arrested human CD4^+^ Th1* cells (**A–C**) and human PBMCs (**D–F**) on non-stimulated (NS: **A**, **D**) and 16–24 h pro-inflammatory cytokine stimulated (**B**, **E** 10 IU/mL TNFα; **C**, **F** 76 IU/mL TNFα + 20 IU/mL IFNγ) human brain microvascular endothelial cells (HBMEC) under flow. Th1* cells or PBMCs were pre-incubated with 1 μg/mL bNTZ and/or with 1 μg/mL of a function-blocking anti-β2-integrin antibody. An isotype control antibody was used as internal control (1 μg/mL Ctrl). **(A-F)** Each figure shows the mean ± SEM of 3 independent experiments. Statistical analysis: one-way ANOVA followed by Tukey’s multiple-comparison test (p < 0.05 = *, p < 0.01 = **, p < 0.001 = ***, p < 0.0001 = ****)

Having titrated the precise concentrations required for NTZ mediated inhibition of T cell interaction with endothelial VCAM-1, we next asked if lack of NTZ mediated inhibition of CD4^+^ T cell arrest under flow was unique to BLEC. Therefore, we next investigated the efficacy of the bNTZ in inhibiting CD4^+^ T cell interaction with the commercially available human brain microvascular endothelial cells (HBMEC). Th1* cells were pre-incubated with 1 μg/mL of bNTZ since this concentration was sufficient to abolish T cell adhesion to immobilized recombinant VCAM-1 under static conditions (Fig. 2A–C). In accordance with our observations with BLEC, bNTZ did not significantly reduce the arrest of Th1* cells to non-stimulated (NS), TNFα or TNFα + IFNγ stimulated HBMEC under physiological flow (Fig. 3A–C). Interestingly, inhibition of β2-integrins on Th1* cells significantly reduced their shear resistant arrest on non-stimulated HBMEC but not on stimulated HBMEC. Significant reduction of Th1* cell arrest to HBMEC was, irrespective of the inflammatory condition, only observed when both, α4- and β2- integrins were blocked (Fig. 3A–C; Additional file 3: Movie S2).

To understand if our observations are limited to CD4^+^ effector/memory T cells, we next explored the interaction of PBMCs with HBMEC under flow. As expected, lower numbers of PBMCs when compared to Th1* cells arrested to NS, TNFα and TNFα + IFNγ stimulated HBMEC (Fig. 3D–F). As already observed for CD4^+^ effector/memory T cells bNTZ failed to significantly reduce the arrest of PBMCs to HBMEC under physiological flow irrespective of their inflammatory condition (Fig. 3D–F). In contrast, inhibition of β2-integrins significantly reduced arrest of PBMCs on NS and TNFα stimulated but not on TNFα + IFNγ stimulated HBMEC (Fig. 3D–F). As already observed for Th1* cells, bNTZ at concentrations sufficient to completely inhibit α4-integrin mediated adhesion to VCAM-1 does not suffice to significantly reduce the arrest of PBMCs on HBMEC under physiological flow.

Taken together, using BLEC and HBMEC as in vitro models of the BBB our observations show that both α4-integrins and β2-integrins can mediate shear resistant arrest of CD4^+^ T cells and PBMCs to the inflamed BBB. Furthermore, as high inflammatory conditions reduced the efficacy of α4- and β2-integrin function-blocking on CD4^+^ T cell arrest to the BBB, T cell arrest on the BBB in the presence of NTZ may be compensated β2-integrin mediated interaction with endothelial ICAM-1, which has been described to mediate post-arrest T cell polarization and crawling against the direction of the blood flow on the BBB [54].

### NTZ fails to inhibit shear resistant T cell arrest to MS patient derived EECM-BMEC-like cells in vitro

**Fig. 4:**
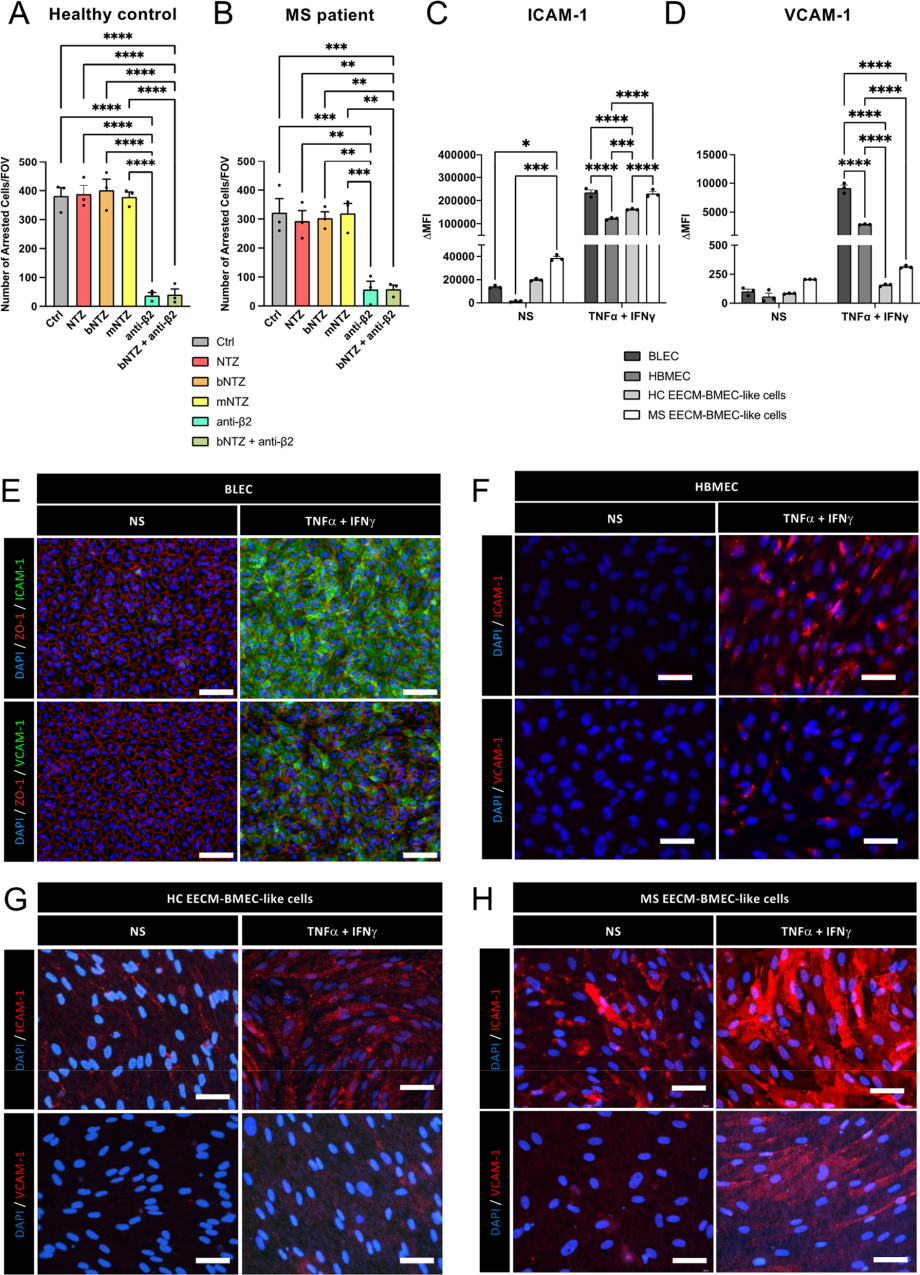
Blocking β2-integrins rather than α4-integrins inhibits shear resistant T cell arrest on EECM-BMEC-like cells expressing high cell surface levels of ICAM-1. Number of arrested human CD4^+^ Th1* cells on 16–24 h pro-inflammatory cytokine stimulated (7.6 IU/mL TNFα + 2 IU/mL IFNγ) EECM-BMEC-like cells derived from a healthy donor (**A**) or an MS patient (**B**) under physiological flow. Th1* cells were preincubated with different natalizumab constructs (1 μg/mL NTZ or 1 μg/mL bNTZ or 1 μg/mL mNTZ) and/or with 1 μg/mL of a function-blocking anti-β2-integrin antibody. An isotype control antibody was used as internal control (1 μg/mL Ctrl). Flow cytometry analysis for the cell-surface expression of ICAM-1 (**C**) and VCAM-1 (**D**) on BLEC, HBMEC and EECM-BMEC-like cells under non-stimulated (NS) and 16 h pro-inflammatory cytokine stimulated conditions (76 IU/mL TNFα + 20 IU/mL IFNγ) are shown. Bar graphs show geometric ΔMFI (MFI specific staining—MFI isotype). **A**–**D** Each figure shows the mean ± SEM of 3 independent experiments. Statistical analysis: one-way ANOVA followed by Tukey’s multiple-comparison test (p < 0.05 = *, p < 0.01 = **, p < 0.001 = ***, p < 0.0001 = ****). Immunofluorescence staining of non-stimulated (NS) and 16 h pro-inflammatory cytokine stimulated (76 IU/mL TNFα + 20 IU/mL IFNγ) BLEC (**E**), HBMEC (**F**) and EECM-BMEC-like cells derived from a healthy donor (**G**) and a MS patient (**H**). **E** Immunostainings for ICAM-1 (green) and VCAM-1 (green) and ZO-1 (red) on BLEC are shown. **F**–**H** Immunostainings for ICAM-1 (red) and VCAM-1 (red) on HBMEC and EECM-BMEC-like cells are shown. **E**–**H** Nuclei were stained with DAPI (blue). Data are representative of 3 independent experiments. Scale bar = **E** 100 μm and **F**–**H** 50 μm

To understand if NTZ inhibits shear resistant arrest of CD4^+^ T cells on in vitro BBB models derived from MS patients, we next made use of hiPSC derived EECM-BMEC-like cells derived from MS patients or healthy controls (HC) [50]. In accordance with our previous findings, we observed that stimulated MS derived EECM-BMEC-like cells supported shear resistant arrest of higher numbers of Th1* cells when compared to HC derived EECM-BMEC-like cells (Fig. 4A, B). Surprisingly, neither 1 μg/mL NTZ, bNTZ nor mNTZ significantly reduced shear resistant arrest of Th1* cells on MS or HC derived EECM-BMEC-like cells under flow (Fig. 4A, B). In contrast, antibody mediated inhibition of β2-integrins significantly reduced shear resistant arrest of Th1* cells to MS and HC derived EECM-BMEC-like cells which was not further reduced by combining bNTZ and the β2-integrin blocking antibody (Fig. 4A, B; Additional file 4: Movie S3).

Taken together, employing three different in vitro models of the human BBB, including one directly derived from MS patients, our data show that the different NTZ constructs failed to significantly reduce T cell arrest on the BBB under physiological flow in vitro. Rather, additional inhibition of β2-integrins was necessary to abrogate T cell arrest on the BBB under physiological flow suggesting a significant role of β2-integrin mediated interaction with endothelial ICAM-1 in our three in vitro BBB models.

### BLEC, HBMEC and EECM-BMEC-like cells display high cell surface expression of ICAM-1 and moderate cell surface expression of VCAM-1

To understand if our three in vitro BBB models may show uniquely high expression levels of ICAM-1 as compared to VCAM-1, we next compared cell surface expression of ICAM-1 and VCAM-1 on non-stimulated and cytokine stimulated BLEC, HBMEC and EECM-BMEC-like cells by flowcytometry. All BBB models showed very low cell surface expression of VCAM-1 while constitutive cell surface expression of ICAM-1 was detected on BLEC and EECM-BMEC-like cells but not on HBMEC (Fig. 4C, D). MS-derived EECM-BMEC-like cells showed the highest constitutive expression of ICAM-1 (Fig. 4C) reflecting their constitutive inflammatory status as described before [50]. Upon stimulation, all in vitro BBB models showed significant cell surface upregulation of ICAM-1 and VCAM-1 (Fig. 4C, D). Stimulated BLEC showed the highest cell surface levels of VCAM-1, accompanied by high levels of ICAM-1. Both, HC and MS EECM-BMEC-like cells showed the lowest cell surface expression of VCAM-1 but under stimulated conditions showed higher ICAM-1 levels when compared to HBMEC (Fig. 4C, D).

Immunofluorescence staining for ICAM-1 and VCAM-1 on confluent monolayers of the different in vitro BBB models (Fig. 4E–H), confirmed our flowcytometry observations, where BLEC and EECM-BMEC-like cells showed the brightest cell surface staining for ICAM-1 under both NS and stimulated conditions. Furthermore, stimulated BLEC showed the strongest surface staining for VCAM-1 compared to the other in vitro BBB models.

Taken together, all in vitro models of the human BBB investigated here displayed constitutive cell surface expression of ICAM-1 rather than VCAM-1 and cytokine stimulation lead to a stronger upregulation of ICAM-1 compared to VCAM-1. Thus, high cell surface levels of ICAM-1 may prohibit NTZ mediated inhibition of CD4^+^ T cell arrest on the BBB under physiological flow in vitro.

### Increasing levels of ICAM-1 impact on α4-integrin T cell mediated interaction with VCAM-1

Having excluded a significant role of α4β1-integrin ligands other than VCAM-1 to T cell arrest we next asked if lack of NTZ mediated inhibition of shear resistant CD4^+^ T cell arrest to the in vitro BBB models could be explained by the high levels of ICAM-1 allowing for β2-integrin mediated T cell arrest in the presence of NTZ. To this end we investigated the arrest of Th1* cells to immobilized recombinant VCAM-1, ICAM-1, and combinations thereof under flow, in the presence and absence of NTZ and/or a function-blocking anti-β2-integrin antibody.

**Fig. 5:**
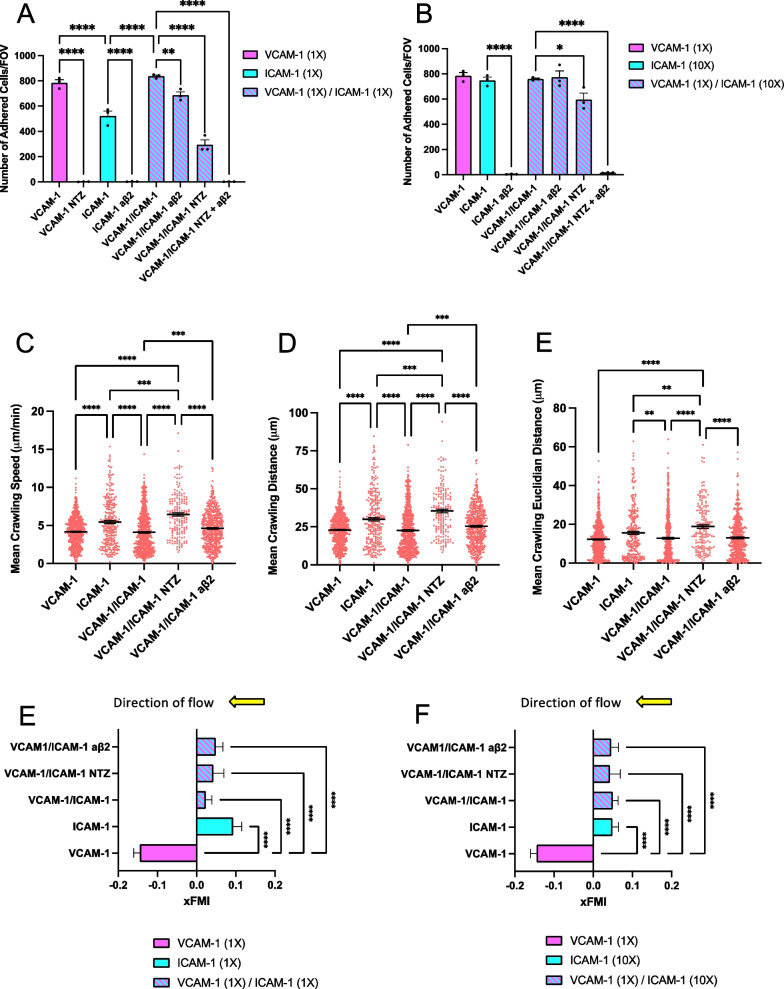
High levels of ICAM-1 compared to VCAM-1 allow for T cell arrest in the presence of natalizumab. **A** Number of arrested human CD4^+^ Th1* cells on equimolar concentrations of immobilized recombinant VCAM-1 (1X, 1.54 μg/mL), ICAM-1 (1X, 1.14 μg/mL), and combined VCAM-1 (1X) / ICAM-1 (1X) under flow condition. **B** Number of adhered Th1* cells on immobilized recombinant VCAM-1 (1X), 10-times more ICAM-1 (10X, 11.4 μg/mL) and combined VCAM-1 (1X) / ICAM-1 (10x) under flow condition. **A**, **B** Th1* cells were treated with 1 μg/mL natalizumab (NTZ) and/or 1 μg/mL of an anti-β2-integrins antibody prior experiment. An isotype control antibody was used as internal control (1 μg/mL Ctrl). Mean crawling speed (**C**), distance (**D**) and Euclidian distance (**E**) of Th1* cells on equimolar concentration of immobilized recombinant VCAM-1 (1X), ICAM-1 (1X), and combined VCAM-1 (1X) / ICAM-1 (1X) under physiological flow conditions are depicted for one representative experiment in each group (> 100 cells per group were analysed). Each data point represents the velocity, the distance, and the Euclidian distance of one cell. **E**, **F** Directionality of Th1* cell crawling on immobilized recombinant VCAM-1, ICAM-1, and combined VCAM-1 / ICAM-1 under physiological flow condition. Results for equimolar concentrations of VCAM-1 (1X) and ICAM-1 (1X) (**E**), and for 10-times more ICAM-1 (10X) are shown (**F**). Directionality of Th1* cell crawling is expressed as xFMI (xFMI = Dx/Dacc, Dx: straight x-axis distance covered by the T cell, Dacc: accumulated total distance of T cell movement). Direction of the physiological flow was along the x-axis from plus to minus and is indicated by an arrow (yellow). **A**, **B**, **E**, **F** Each figure shows the mean ± SEM of 3 independent experiments. Statistical analysis: one-way ANOVA followed by Tukey’s multiple-comparison test. **C**, **D **Each figure shows the mean ± SEM of one representative experiment per group (> 100 cells per condition were analysed). Statistical analysis: one-way ANOVA followed by Dunn’s multiple-comparison test (Kruskal–Wallis test) (p < 0.05 = *, p < 0.01 = **, p < 0.001 = ***, p < 0.0001 = ****)

Comparing the shear resistant arrest of Th1* cells to reducing concentrations of VCAM-1 and ICAM-1 showed a significant and dose-dependent reduction of shear resistant Th1* arrest to VCAM-1 and ICAM-1 (Additional file 1: Fig. S3A, B). Significantly higher numbers of Th1* cells arrested on equimolar concentrations of VCAM-1 than ICAM-1 and these interactions were completely abolished by NTZ and the function blocking anti-β2-integrin antibody, respectively (Fig. 5A; Additional file 5: Movie S4). A tenfold higher molar concentration of ICAM-1 compared to VCAM-1 was necessary to achieve comparable numbers of Th1* cells arresting under flow in vitro which was again completely abrogated by pretreating Th1* cells with the anti-β2-integrin antibody (Fig. 5B). Thus, Th1* cells preferentially arrest on VCAM-1 over ICAM-1. Combining next equimolar amounts of VCAM-1 and ICAM-1 or of VCAM-1 with tenfold ICAM-1 over VCAM-1 did not further enhance shear resistant Th1* cell arrest under flow when compared to VCAM-1 alone (Fig. 5A, B). Blocking β2-integrins did slightly reduce Th1* cell arrest to equimolar combinations of VCAM-1/ICAM-1 when compared to VCAM-1 but did not affect Th1* cell arrest to combinations of VCAM-1/tenfold ICAM-1 compared to VCAM-1. Interestingly, NTZ reduced the number of Th1* cells arrested on VCAM-1/ICAM-1 compared to VCAM-1 almost fourfold while still significantly reducing Th1* cells arrested on VCAM-1/tenfold ICAM-1 compared to VCAM-1. Complete abrogation of Th1* cell arrest to VCAM-1/ICAM-1 under physiological flow could only be achieved by combined blocking of both, α4- and β2-integrins (Fig. 5A, B). Collectively, these observations show that although Th1* cells preferentially arrest on VCAM-1, increasing concentrations of ICAM-1 reduce efficacy of NTZ mediated inhibition of their arrest under physiological flow.

To advance our understanding of the differential role of ICAM-1 and VCAM-1 in CD4^+^ T cell arrest and post-arrest behavior on the BBB, we next investigated the mean crawling speed, distance, and Euclidian distance, as also the directionality of Th1* cells on immobilized recombinant VCAM-1, ICAM-1, and combinations thereof under flow conditions in the presence and absence of NTZ and/or a function-blocking anti-β2-integrin antibody. The mean crawling speed and distance of Th1* cells on VCAM-1 were significantly lower compared to those on equimolar concentrations of ICAM-1, while equimolar concentrations of VCAM-1/ICAM-1 reduced T cell crawling speeds and distance to those observed on equimolar concentrations of VCAM-1 (Fig. 5C, D). As expected, in the presence of NTZ the mean crawling speed and distance of Th1* cells on VCAM-1/ICAM-1 was significantly increased when compared to untreated conditions (Fig. 5C, D). Surprisingly however, β2-integrin blocking also significantly increased the mean crawling speed and distance of Th1* cells on VCAM-1/ICAM-1, when compared to VCAM-1, although the effect was only minor when compared to NTZ treated Th1* cells (Fig. 5C, D). Interestingly, the mean crawling speed and distance of Th1* cells on tenfold higher ICAM-1 were comparable to these observed on VCAM-1 (Additional file 1: Fig. S4A, B) and significantly lower when compared to onefold ICAM-1 (Fig. 5C, D) underscoring that the overall avidity of adhesive interactions controls Th1* crawling speed and distance. NTZ did not significantly increase the mean crawling speed and distance of Th1* cells on VCAM-1 combined with tenfold ICAM-1, when compared to tenfold ICAM-1 alone (Additional file 1: Fig. S4A, B). However, blocking β2-integrins significantly increased the mean crawling speed and distance of Th1* cells on VCAM-1 combined with tenfold ICAM-1 when compared to untreated conditions. The observation that the crawling behavior of Th1* cells does not show a Gaussian distribution and that combinations of ICAM-1 and VCAM-1 impacted not only on the overall Th1* crawling speed but also on the pattern of crawling of the individual cells should be noted. Analysis of the mean crawling Euclidian distance was in line with the results obtained for the mean crawling distance of Th1* cells on VCAM-1, ICAM-1, and combinations thereof for both, equimolar and 10-times more ICAM-1 conditions (Fig. 5E; Additional file 1: Fig. S4C). In addition, we observed a role for shear stress on the movement of Th1* cells on VCAM-1, ICAM-1 and combinations thereof. We specifically observed that Th1* cells arrested on VCAM-1 had a significantly different crawling directionality (xFMI) compared to Th1* cells interacting with ICAM-1 or combined VCAM-1/ICAM-1 in both equimolar (Fig. 5E) and tenfold ICAM-1 conditions (Fig. 5F). Specifically, while in the presence of ICAM-1 Th1* cells resisted the shear stress and crawled against the direction of flow, they rather crawled in the direction of flow on VCAM-1 (Additional file 1: Fig. S4D–F; Additional file 6: Movie S5).

Taken together, these results underscore that human CD4^+^ T cells behave differently on isolated VCAM-1 or ICAM-1 and combinations thereof under physiological flow in vitro.

### Dose-dependent inhibition of NTZ on T cell arrest to VCAM-1 under flow is abrogated by high levels of ICAM-1

**Fig. 6:**
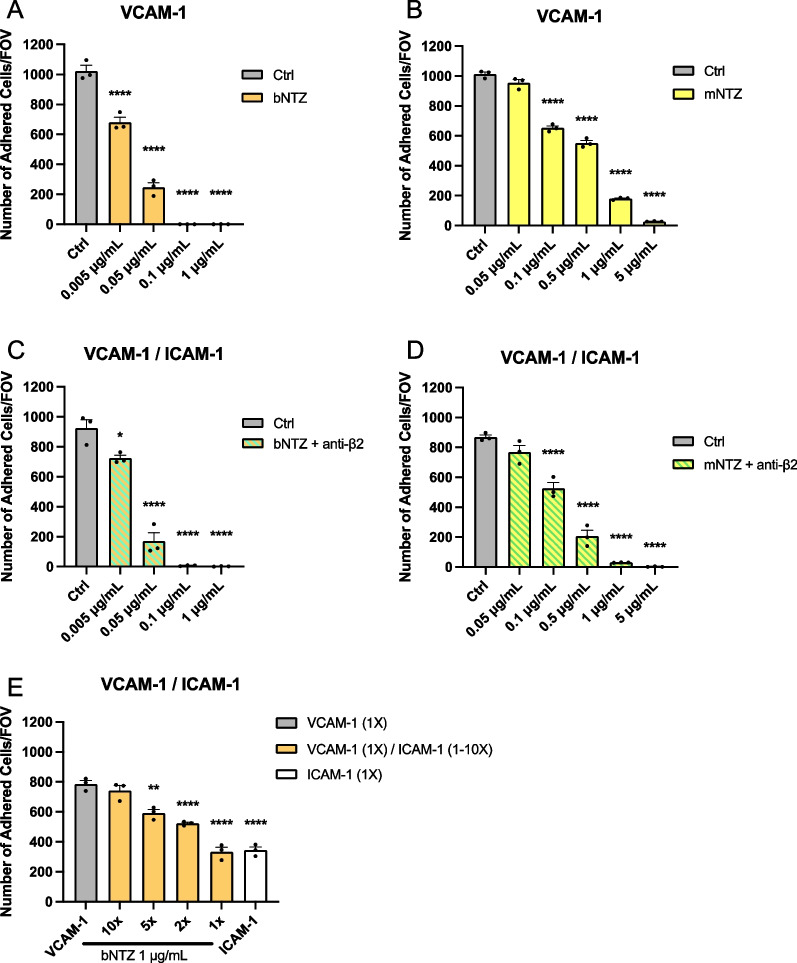
Dose dependent inhibition of shear resistant T cell arrest to VCAM-1 is abrogated in the presence of high levels of ICAM-1. Number of arrested human CD4^+^ Th1* cells on immobilized recombinant VCAM-1 (1X, 1.54 μg/mL) (**A**, **B**), and equimolar concentrations of VCAM-1 (1X) / ICAM-1 (1X, 1.14 μg/mL) combined (**C**, **D**) under flow conditions. Th1* cells were treated with titrated concentrations of the different natalizumab constructs as indicated (bNTZ: **A**, **C**; mNTZ: **B**, **D**) and/or with 1 μg/mL of a function blocking anti-β2-integrin antibody (**C**, **D**) prior experiment. An isotype control antibody was used as internal control (1 μg/mL Ctrl). **E** Number of arrested human Th1* cells on equimolar concentration of immobilized recombinant VCAM-1 (1X) and ICAM-1 (1X), and VCAM-1 (1X) / ICAM-1 (1-10X, 1.14–11.4 μg/mL) combined under flow condition. Th1* cells arresting to immobilized recombinant VCAM-1/ICAM-1 combined were treated with 1 μg/mL bNTZ prior experiment. **A**–**E** Each figure shows mean ± SEM of 3 independent experiments. Statistical analysis: unpaired T-test (p < 0.05 = *, p < 0.01 = **, p < 0.001 = ***, p < 0.0001 = ****). Significant differences are shown only for the comparison with Ctrl condition

As NTZ undergoes Fab-arm exchange in vivo, we finally aimed to understand the dose dependent effects of bNTZ and mNTZ on inhibiting the arrest of Th1* cells to immobilized recombinant VCAM-1 under physiological flow in vitro. In accordance with our observations under static conditions (Fig. 2A–C), we observed a dose dependent inhibition of bNTZ and mNTZ on Th1* arrest to VCAM-1 under flow conditions (Fig. 6A, B). While bNTZ showed a minimal significant inhibitory concentration of 0.005 μg/mL, mNTZ exerted its minimal significant inhibitory concentration at 0.1 μg/mL, confirming the more potent inhibitory activity of bNTZ compared to mNTZ also under flow conditions. Similarly, bNTZ inhibited Th1* arrest to VCAM-1 with an efficiency higher than 95% at a concentration of 0.1 μg/mL when compared to the 5 μg/mL required for mNTZ (Additional file 1: Fig. S5A, B).

Assessing next the efficacy of both NTZ constructs on inhibiting Th1* cell arrest to equimolar concentrations of VCAM-1 and ICAM-1 under flow, we found that both dose dependently inhibited the arrest of Th1* cells to the equimolar combination of VCAM-1 and ICAM-1, when β2-integrins were blocked (Fig. 6C, D). Furthermore, the minimal and maximal inhibitory concentrations of bNTZ and mNTZ required to inhibit Th1* arrest to equimolar coatings of VCAM-1 and ICAM-1 were identical to those determined for inhibiting Th1* arrest on VCAM-1 alone (Fig. 6A–D and Additional file 1: Fig. S5A–D), suggesting a 20 to 50 higher efficacy of bNTZ compared to the mNTZ. In sum, these data show that both NTZ constructs are able to inhibit Th1* arrest to VCAM-1 in the presence of equimolar concentrations of ICAM-1 under physiological flow conditions in a comparable manner as previously observed under static conditions.

To finally understand which levels of ICAM-1 may impact on efficacy of NTZ mediated inhibition of T cell arrest on the BBB, we investigated Th1* arrest under physiological flow on combinations of immobilized recombinant VCAM-1 with equimolar (1X) or increasing molar concentrations (2X, 5X, 10X) of ICAM-1. Increasing the concentration of ICAM-1 over VCAM-1 increased the number of Th1* cells able to undergo shear resistant arrest under physiological flow despite the presence of bNTZ (Fig. 6E). While on equimolar concentrations of ICAM-1 and VCAM-1, 1 μg/ml NTZ reduced Th1*cell arrest by 50%, in conditions of tenfold higher ICAM-1 versus VCAM-1 this concentration of NTZ failed to reduce Th1* arrest under physiological flow (Fig. 6E).

Taken together, these observations underscore that NTZ efficiently inhibits α4-integrin mediated arrest on VCAM-1 under flow. At the same time, we here demonstrate that high levels of ICAM-1 compared to VCAM-1 abrogated NTZ mediated inhibition of CD4^+^ T cell arrest to ICAM-1 and VCAM-1 coated surfaces.

## Discussion

The present study confirms that NTZ fully abrogates α4-integrin-mediated CD4^+^ T cell interaction with endothelial VCAM-1. By titrating bNTZ and mNTZ, we defined the minimal and the maximal inhibitory concentration required for each NTZ construct to reduce shear resistant arrest of CD4^+^ T cell on VCAM-1. In apparent contrast to these observations, all NTZ constructs failed to efficiently inhibit the arrest of CD4^+^ T cells and PBMCs on three different human in vitro BBB models under physiological flow. As we identified high surface levels of ICAM-1 expressed on the human BBB models, we investigated the interaction of CD4^+^ T cells on combinations of VCAM-1 and ICAM-1 under flow. Although CD4^+^ T cells more avidly bound to VCAM-1 compared to ICAM-1, tenfold higher molecular concentrations of ICAM-1 than VCAM-1 abrogated NTZ mediated inhibition of CD4^+^ T cell arrest. Accordingly, abrogation of CD4^+^ T cell or PBMC arrest on the in vitro BBB models under flow could only be achieved by combined blocking of α4- and β2-integrins. NTZ mediated inhibition of CD4^+^ T cell interaction with the BBB in MS may thus be hampered by high ICAM-1 expression levels on the BBB and should be taken into account when considering safety of EID of NTZ.

BLEC and hiPCS-derived EECM-BMEC-like cells represent in vitro BBB models that establish barrier properties comparable to primary human brain microvascular endothelial cells [70] with mature tight junctions and low permeability to small molecular tracers [47, 49]. With respect to barrier properties BLECs and EECM-BMEC-like cells are thus superior to the commercially available HBMEC [71], also used in this study, which show lower trans-endothelial electrical resistance (TEER) and higher permeability to small molecules [72]. Upon stimulation with pro-inflammatory cytokines, BLECs as well as EECM-BMECs develop impaired barrier properties, upregulate ICAM-1 and VCAM-1, and support multi-step immune cell extravasation under physiological flow [44, 50, 73], thus mimicking closely the situation of neuroinflammation in vivo [47–49].

Aiming to compare the efficacy of bivalent and monovalent NTZ constructs in inhibiting T cell interaction with the BBB in vitro*,* we here made the surprising observation that neither NTZ, bNTZ, nor mNTZ significantly inhibited CD4^+^ T cell arrest to stimulated BLEC, HBMEC, or EECM-BMEC-like cells under flow conditions. These results are in apparent contrast to our previous observations, which demonstrated that α4-integrins mediate the shear-resistant arrest of encephalitogenic mouse CD4^+^ T cells on a mouse in vitro BBB model [74] or the arrest of mouse or human CD4 T cells to mouse spinal cord microvascular endothelial cells in vivo [75, 76]. Post-arrest T cell polarization on the BBB and crawling against the direction of blood-flow to sites permissive for diapedesis is subsequently mediated by β2-integrins interacting with endothelial ICAM-1 and ICAM-2 [77]. These previous studies differ from the present study by examining CD4^+^ T cell interactions with the BBB of the mouse and investigation of different CD4^+^ T cell subsets. To determine potential differences in expression levels of ICAM-1 and VCAM-1 in the mouse and the human BBB we made use of accessible datasets of a side-by-side comparison of the transcriptome profiles of brain microvessels dissected from C57BL/6 mice and normal human brain tissue obtained during neurosurgery [58]. These data show that human brain microvessels express about threefold higher ICAM-1 levels when compared to VCAM-1 (analysis workflow described in Methods). In contrast, in mouse brain microvessels VCAM-1 expression levels were found to be twofold higher than those of ICAM-1. These data suggest that ICAM-1 levels at the human BBB may be higher than those observed in the mouse. In accordance with these findings, we here show that cytokine stimulation resulted in a stronger upregulation of ICAM-1 compared to VCAM-1 on all three human in vitro BBB models. We therefore hypothesized that high levels of endothelial ICAM-1 may mask NTZ mediated inhibition of α4β1-integrin mediated CD4^+^ T cell arrest to VCAM-1 on the BBB by providing an alternative mechanism for T cell interaction with the BBB. In line with this, additional blocking of β2-integrins in the presence of NTZ significantly reduced CD4^+^ T cell arrest to the inflamed BBB under flow in vitro*,* while tenfold higher molecular concentration of ICAM-1 compared to VCAM-1 allowed for T cell arrest under flow in vitro in the presence of NTZ.

Our present study highlights the different roles of ICAM-1 and VCAM-1 in the interaction with human effector/memory CD4^+^ T cells. Higher numbers of CD4^+^ T cells arrested on equimolar concentrations of VCAM-1 compared to ICAM-1 underscoring an overall higher avidity of CD4^+^ T cells to VCAM-1. In accordance to previous observations, CD4^+^ T cells could arrest on ICAM-1 and VCAM-1 under physiological flow [54, 78], but presence of ICAM-1 was required to initiate T cell crawling against the direction of flow. These observations confirm that α4β1-integrin-VCAM-1 interactions mediate shear resistant arrest of CD4^+^ T cells to the BBB [74–76], while post-arrest behavior of CD4^+^ T cells on the BBB is rather mediated by αLβ2-integrin engaging endothelial ICAM-1 and ICAM-2 [54, 78]. Indeed, our present study confirmed that blocking β2-integrins but not α4-integrins impacted on post-arrest CD4^+^ T cell interaction with the BBB.

Here we found that very high levels of endothelial ICAM-1 may prohibit efficient NTZ mediated abrogation of α4β1-integrin mediated CD4^+^ T cell arrest to the BBB in neuroinflammation. Unfortunately, treatment with efalizumab, a humanized IgG1 monoclonal antibody targeting αL-integrin, is also associated with the risk of PML in patients with psoriasis [79, 80]. Therefore, targeting αL-integrin-ICAM-1 interactions in MS patients may also increase the risk for PML.

Although to our knowledge there is no study directly comparing ICAM-1 and VCAM-1 expression levels on the BBB during RRMS, increased levels of the soluble form of ICAM-1 (sICAM-1) and VCAM-1 (sVCAM-1) in the CSF and serum have been reported in MS patients with higher disease activity [81–83]. Thus, comparative analysis of sICAM-1 and sVCAM-1 serum levels in RRMS patients with clinical and radiological activity during NTZ treatment may be useful to anticipate the effectiveness of NTZ treatment and to explore if MS patients showing higher levels of sICAM-1 than sVCAM-1 may be less responsive to NTZ treatment.

Therapeutic efficacy of blocking α4-integrins to inhibit CD4^+^ T cell migration across the BBB was discovered in EAE, why in the present study we focused on CD4^+^ T cells. However, there is substantial evidence implicating CD8^+^ T cells in MS, as CD8^+^ T cells outnumber CD4^+^ T cells in demyelinating lesions of MS patients [84, 85]. Furthermore, predominant infiltration of CD8^+^ T cells and close proximity to JCV-infected glial cells is observed in brain biopsies of patients with PML [86, 87]. Thus, additional studies are needed to assess the dose dependent effect of NTZ on CD8^+^ T cell interactions with the BBB and to understand if NTZ might have differential effects on CD8^+^ versus CD4^+^ T cell migration across the BBB.

Since NTZ undergoes “Fab-arm exchange” with other IgG4 antibodies in vivo [37–39], we here performed a side-by-side comparison of the dose-dependent effects of bivalent or monovalent NTZ constructs in inhibiting α4β1-integrin mediated interactions with VCAM-1 under physiological flow conditions. We observed a minimal inhibitory concentration of 0.005 μg/mL and 0.01 μg/mL for bNTZ and mNTZ, respectively, and a maximal inhibitory concentration of 0.1 μg/mL and 5 μg/mL for bNTZ and mNTZ respectively. The latter is much lower when compared to the NTZ concentrations measured in RRMS patients with SID of NTZ at time of redosing, which usually ranges from 25 to 35 μg/mL [88–90], but is closer, especially for the mNTZ construct, to the average concentrations of 10.8 μg/mL and 18.2 μg/mL measured in patients with EID in two different studies [89, 90]. Our data therefore suggest that EID of NTZ can maintain its therapeutic effect in inhibiting T cell adhesion to VCAM-1 on the BBB in MS patients. In accordance to our present findings, plasma concentrations of NTZ between 1 and 2 μg/mL were shown sufficient in most patients to reach consistent saturation of α4-integrins, with a receptor occupancy on PBMCs above 80% [91], while serum concentrations below 1 μg/mL lead to desaturation of α4-integrins and a receptor occupancy below 50% on PBMCs [91]. Combined with these previous observations our results suggest that EID (6 weeks interval) of NTZ might suffice to block α4-integrin mediated T cell arrest to the BBB. In addition, our study alludes to the possibility that EID may be even extended beyond the 6 weeks interval, since we observed that mNTZ concentrations lower than those measured in EID-NTZ patients at time of redosing are still effective in inhibiting the interaction of CD4^+^ T cells with VCAM-1 in vitro. Besides EID of NTZ, the body mass index was described as an additional variable influencing the effectives and pharmacodynamics of NTZ [92] which was not directly addressed in the present study. However, we here showed that conditions raising expression levels of ICAM-1 at the BBB may abrogate effectiveness of NTZ mediated inhibition of T cell interaction with the BBB. Thus, high body mass index, which often correlates with obesity (and thus elevated systemic inflammation), may be a condition where caution should be used when determining the dosing interval for MS patients.

Taken together, our study shows that NTZ is highly effective in inhibiting α4-integrin mediated CD4^+^ T cell interaction with VCAM-1 on the BBB in vitro at doses that are even lower than those observed in the serum of patients undergoing EID-NTZ treatment. While this may indicate the safety of EID of NTZ in MS, we observed at the same time that high expression levels of ICAM-1 on the BBB in vitro may abrogate the therapeutic effects of NTZ. Our in vitro observations therefore could be interpreted such that the therapeutic efficacy of NTZ may rely on inhibiting the migration of autoaggressive CD4^+^ T cells across the BBB under low inflammatory conditions, e.g. during remission, when ICAM-1 expression levels on the BBB are low or moderate. At the same time elevated levels of ICAM-1 on the BBB and high numbers of CNS infiltrating CD8^+^ T cells are observed in the brains of patients with PML [86, 87] arguing that NTZ may not interfere with T cell mediated CNS immune defense against PML. However, elevated ICAM-1 levels on the BBB may be secondary to JCV reactivation and virus-related CNS damage allowing in the presence of NTZ for ICAM-1 mediated CD8^+^ T cell recruitment into the CNS only at later timepoints when JCV induced inflammation has already progressed and ICAM-1 levels on the BBB have sufficiently increased.

Considering that the conclusions we can draw are still speculative, it is mandatory, to further explore which patients may specifically benefit from EID of NTZ, further studies will be needed, including exploring the options for determining expression levels of ICAM-1 and VCAM-1 on the BBB in MS patients. To this end, translating methodologies from mouse models of MS that have allowed to image the inflammatory status of the BBB in vivo by MRI imaging making use of micro-sized particles of iron oxide (MPIO) targeting adhesion molecules including ICAM-1 [93, 94], will be beneficial to further advance the personalized dosing of NTZ in individual MS patients ensuring therapeutic efficacy while limiting the risk for PML.

## Supplementary Information

**Additional file 1: Figure S1.** Flow cytometry analysis of Th1* cell-bound NTZ, bNTZ, mNTZ, and anti-β2-antibody at different concentrations: 50 μg/mL, 10 μg/mL, 1 μg/mL, 0.1 μg/mL, 0.01 μg/mL, and 0.001 μg/mL. Isotype controlis shown in grey. ΔMFI is indicated next to each peak of detection. Percentage of human CD4^+^ Th1 adhesion inhibited by titrated NTZ , bNTZ and mNTZ to immobilized recombinant VCAM-1 under static conditions. Red dotted line shows 95% limit of T cell adhesion inhibition. Number of adhered Th1/Th1*/Th2/Th17 cells on immobilized recombinant VCAM-1 under static conditions grouped by treatment condition. Th1/Th1*/Th2/Th17 cells were treated with the minimal inhibitory concentration shown in Fig. 2 –C for the respective natalizumab constructsprior to the experiment. An isotype control antibody was used as internal control. Each figure shows the mean ± SEM of 3 experiments. Gating strategy for the multicolor flow cytometry analysis of α4-, β1- and β7-integrin cell-surface expression on Th1, Th1*, Th2and Th17 cells. Isotype control condition is shown in grey. Percentage and ΔMFI of α4-, β1- and β7-integrins high and low expressing Th1, Th1*, Th2and Th17 cellsis shown. **Figure S2.** Number of adhered human effector/memory CD4^+^ Th1* cells on immobilized recombinant JAM-B and fibronectin under static conditions . Th1* cells were incubated with 1 μg/mL natalizumabor with 1 μg/mL of an isotype control antibodyprior experiment. Each figure shows the mean ± SD of 2 independent experiments done in triplicates. Statistical analysis: unpaired T-test. **Figure S3.** Number of adhered human CD4^+^ Th1* cells on titrated concentrations of immobilized recombinant VCAM-1 and ICAM-1 under flow conditions. Each figure shows the mean ± SEM of 3 independent experiments. **Figure S4.** Mean crawling speed , distance and Euclidian distance of human CD4^+^ Th1* cells on immobilized recombinant VCAM-1, 10-times higher molecular concentrations of ICAM-1and combined VCAM-1/ ICAM-1under physiological flow condition. Th1* cells were treated with 1 μg/mL natalizumab and/or with 1 μg/mL of functional anti-β2-integrins blocking antibody prior experiment. An isotype control antibody was used as internal control. Each figure shows the mean ± SEM of one representative experiment per group. Statistical analysis: one-way ANOVA followed by Dunn’s multiple-comparison test. x/y diagrams of Th1*-cell crawling tracks on immobilized recombinant VCAM-1, ICAM-1, and combined VCAM-1/ ICAM-1 under physiological flow conditions are depicted for one representative experiment per group. For each track, the site of arrest was set to the center point of the respective diagram. End points of tracks are indicated by dots. Flow direction is illustrated by an arrow. **Figure S5.** Percentage of human CD4^+^ Th1* adhesion inhibited by titrated bNTZ and mNTZ to immobilized recombinant VCAM-1, and combined VCAM-1/ ICAM-1when β2-integrins are blocked under flow condition. Red dotted line shows 95% limit of T cell adhesion inhibition. Each figure shows the mean ± SEM of 3 experiments.

**Additional file 2: Movie S1.** Representative time lapse videos of human CD4^+^ Th1* interaction on TNFα/IFNγ stimulated BLEC under physiological flow. Th1* cell treatment conditions with isotype control antibody, bivalent natalizumab, monovalent natalizumab, and bNTZ + anti-β2-integrin blocking antibody are shown respectively from the left to the right. 14 min of recording are shown: 4 min of accumulation phaseand 10 min of physiological shear stress. Phase contrast channel is shown. Time is indicated as minutes:seconds. Flow direction is illustrated by an arrow. Scale bar = 100 μm.

**Additional file 3: Movie S2.** Representative time lapse videos of human CD4^+^ Th1* interaction on TNFα/ IFNγ stimulated HBMEC under physiological flow. Th1* cells were prelabelled with CMFDA prior to the experiment. Th1* cell treatment conditions with isotype control antibody, bivalent natalizumab, and bNTZ + anti-β2-integrin blocking antibody are shown from the top to the bottom, respectively. 9 min of recording are shown: 4 min of accumulation phase and 5 min of physiological shear stress. Phase contrast and green fluorescent channels are overlayed. Time is indicated as minutes:seconds. Flow direction is illustrated by an arrow. Scale bar = 100 μm.

**Additional file 4: Movie S3.** Representative time lapse videos of human CD4^+^ Th1* interaction on TNFα/IFNγ stimulated EECM-BMEC-like cells from a healthy donor under physiological flow. Th1* cells were prelabelled with CMFDA prior to the experiment. Th1* cell treatment conditions with isotype control antibody, bivalent natalizumab, and anti-β2-integrin blocking antibody are shown from the top to the bottom, respectively. 9 min of recording are shown: 4 min of accumulation phase and 5 min of physiological shear stress. Phase contrast and green fluorescent channels are overlayed. Time is indicated as minutes:seconds. Flow direction is illustrated by an arrow. Scale bar = 100 μm.

**Additional file 5: Movie S4.** Representative time lapse videos of human CD4^+^ Th1* interaction on equimolar concentrations of immobilized recombinant ICAM-1, VCAM-1, and combinations of both under physiological flow. Th1* cells were prelabelled with CMFDA prior to the experiment. Th1* cell treatment conditions with isotype control antibody, anti-β2-integrin blocking antibody, natalizumab and NTZ + anti-β2-integrin blocking antibody are shown from the top to the bottom on the right side, respectively. 9 min of recording are shown: 4 min of accumulation phase and 5 min of physiological shear stress. Phase contrast and green fluorescent channels are overlayed. Time is indicated as minutes:seconds. Flow direction is illustrated by an arrow. Scale bar = 200 μm.

**Additional file 6: Movie S5.** Representative time lapse videos of x/y diagrams of human CD4^+^ Th1* crawling tracks on equimolar concentrations of immobilized recombinant ICAM-1, VCAM-1, and combinations of both under physiological flow. Crawling tracks over 5 min of physiological shear stress are shown. For each track, the site of arrest was set to the center point of the respective diagram. End points of tracks are indicated by a dot. Time is indicated as minutes:seconds. Flow direction is illustrated by an arrow.

**Additional file 7: Table S1.** Antibody list for fluorescence-activated cell sorting of different CD4^+^ Th subsets. **Table S2.** Antibody list for flowcytometry analysis of adhesion molecules surface expression of BLEC, HBMEC and EECM-BMEC-like cells. **Table S3.** Antibody list for flowcytometry analysis of integrins surface expression of different CD4^+^ Th subsets. **Table S4.** Antibody list for immunofluorescence staining of adhesion molecules surface expression of BLEC, HBMEC and EECM-BMEC-like cells.

## Data Availability

Data—not applicable; materials are commercially available or can be made available upon request to the corresponding author.

## Abbreviations

BBB: Blood–brain barrier
BLEC: Brain-like endothelial cells
BMEC: Brain microvascular endothelial cells
CD: Cluster of differentiation
CNS: Central nervous system
DPBS: Dulbecco's phosphate-buffered saline
EAE: Experimental autoimmune encephalomyelitis
ECM: Endothelial cell medium
EECM: Extended endothelial-cell culture method
EID: Extended interval dosing
FACS: Fluorescence-activated cell sorting
FBS: Fetal bovine serum
FOV: Field of view
HBMEC: Human brain microvascular endothelial cells
ICAM-1: Intercellular adhesion molecule 1
IFNγ: Interferon-γ
IgG: Immunoglobulin G
IL: Interleukin-2
IU: International unit
JAM-B: Junctional adhesion molecule B
JCV: John Cunningham virus
MAM: Migration assay medium
MS: Multiple sclerosis
NPN: Nano-porous nitrite
NTZ: Natalizumab
PBMC: Peripheral blood mononuclear cells
PC membrane: Polycarbonate membrane
PML: Progressive multifocal leukoencephalopathy
PSGL-1: P-selectin glycoprotein ligand 1
RRMS: Relapsing–remitting MS
SID: Standard interval dosing
Th: T helper
TNFα: Tumor necrosis factor-α
VCAM-1: Vascular cell-adhesion molecule 1
VEGF: Vascular endothelial growth factor
VLA-4: Very late antigen 4
ZO-1: Zonula occludens 1
