# Supplementary material for: High levels of endothelial ICAM-1 prohibit natalizumab mediated abrogation of CD4+ T cell arrest on the inflamed BBB under flow in vitro
Source: J Neuroinflammation. 2023 May 23;20:123. doi: 10.1186/s12974-023-02797-8 (PMC10204262; doi:10.1186/s12974-023-02797-8)
Supplement: Supplementary file 1 — Additional file 1: Figure S1. Flow cytometry analysis of Th1* cell-bound NTZ, bNTZ, mNTZ, and anti-β2-antibody at different concentrations: 50 μg/mL, 10 μg/mL, 1 μg/mL, 0.1 μg/mL, 0.01 μg/mL, and 0.001 μg/mL. Isotype controlis shown in grey. ΔMFI is indicated next to each peak of detection. Percentage of human CD4+ Th1 adhesion inhibited by titrated NTZ , bNTZ and mNTZ to immobilized recombinant VCAM-1 under static conditions. Red dotted line shows 95% limit of T cell adhesion inhibition. Number of adhered Th1/Th1*/Th2/Th17 cells on immobilized recombinant VCAM-1 under static conditions grouped by treatment condition. Th1/Th1*/Th2/Th17 cells were treated with the minimal inhibitory concentration shown in Fig. 2 –C for the respective natalizumab constructsprior to the experiment. An isotype control antibody was used as internal control. Each figure shows the mean ± SEM of 3 experiments. Gating strategy for the multicolor flow cytometry analysis of α4-, β1- and β7-integrin cell-surface expression on Th1, Th1*, Th2and Th17 cells. Isotype control condition is shown in grey. Percentage and ΔMFI of α4-, β1- and β7-integrins high and low expressing Th1, Th1*, Th2and Th17 cellsis shown. Figure S2. Number of adhered human effector/memory CD4+ Th1* cells on immobilized recombinant JAM-B and fibronectin under static conditions . Th1* cells were incubated with 1 μg/mL natalizumabor with 1 μg/mL of an isotype control antibodyprior experiment. Each figure shows the mean ± SD of 2 independent experiments done in triplicates. Statistical analysis: unpaired T-test. Figure S3. Number of adhered human CD4+ Th1* cells on titrated concentrations of immobilized recombinant VCAM-1 and ICAM-1 under flow conditions. Each figure shows the mean ± SEM of 3 independent experiments. Figure S4. Mean crawling speed , distance and Euclidian distance of human CD4+ Th1* cells on immobilized recombinant VCAM-1, 10-times higher molecular concentrations of ICAM-1and combined VCAM-1/ ICAM-1under physiolo [file 12974_2023_2797_MOESM1_ESM.docx]

**Supplementary Figures**

**Figure S1.**

**(A)** Flow cytometry analysis of Th1* cell-bound NTZ, bNTZ, mNTZ, and anti-b2-antibody at different concentrations (from top to bottom): 50 μg/mL, 10 μg/mL, 1 μg/mL, 0.1 μg/mL, 0.01 μg/mL, and 0.001 μg/mL. Isotype control (Ctrl) is shown in grey. ΔMFI is indicated next to each peak of detection. Percentage of human CD4^+^ Th1 adhesion inhibited by titrated NTZ **(B)**, bNTZ **(C)** and mNTZ **(D)** to immobilized recombinant VCAM-1 under static conditions. Red dotted line shows 95% limit of T cell adhesion inhibition. **(E)** Number of adhered Th1/Th1*/Th2/Th17 cells on immobilized recombinant VCAM-1 under static conditions grouped by treatment condition. Th1/Th1*/Th2/Th17 cells were treated with the minimal inhibitory concentration shown in Figure 2 A-C for the respective natalizumab constructs (0.01 μg/mL NTZ or 0.01 μg/mL bNTZ or 0.1 μg/mL mNTZ) prior to the experiment. An isotype control antibody was used as internal control (0.01 or 0.1 μg/mL Ctrl). **(B-E)** Each figure shows the mean ± SEM of 3 experiments. **(F)** Gating strategy for the multicolor flow cytometry analysis of α4 -, β1- and β7-integrin cell-surface expression **(G)** on Th1 (green), Th1* (red), Th2 (blue) and Th17 cells (orange). Isotype control condition is shown in grey. Percentage **(H)** and ΔMFI **(I)** of α4-, β1- and β7-integrins high and low expressing Th1 (green), Th1* (red), Th2 (blue) and Th17 cells (orange) is shown.

**Figure S2.** Number of adhered human effector/memory CD4^+^ Th1* cells on immobilized recombinant JAM-B **(A)** and fibronectin under static conditions **(B)**. Th1* cells were incubated with 1 μg/mL natalizumab (NTZ) or with 1 μg/mL of an isotype control antibody (Ctrl) prior experiment. Each figure shows the mean ± SD of 2 independent experiments done in triplicates. Statistical analysis: unpaired T-test (p < 0.05 = *, p < 0.01 = **, p < 0.001 = ***, p < 0.0001 = ****).

**Figure S3.** Number of adhered human CD4^+^ Th1* cells on titrated concentrations of immobilized recombinant VCAM-1 **(A)** and ICAM-1 **(B)** under flow conditions. **(A, B)** Each figure shows the mean ± SEM of 3 independent experiments.

**Figure S4.** Mean crawling speed **(A)**, distance **(B)** and Euclidian distance **(C)** of human CD4^+^ Th1* cells on immobilized recombinant VCAM-1 (1X), 10-times higher molecular concentrations of ICAM-1 (10X, 11.4 μg/mL) and combined VCAM-1 (1X) / ICAM-1 (10X) under physiological flow condition. Th1* cells were treated with 1 μg/mL natalizumab (NTZ) and/or with 1 μg/mL of functional anti-β2-integrins blocking antibody prior experiment. An isotype control antibody was used as internal control (1 μg/mL Ctrl). **(D, E)** Each figure shows the mean ± SEM of one representative experiment per group (>100 cells per condition were analysed). Statistical analysis: one-way ANOVA followed by Dunn’s multiple-comparison test (Kruskal-Wallis’s test) (p < 0.05 = *, p < 0.01 = **, p < 0.001 = ***, p < 0.0001 = ****). x/y diagrams of Th1*-cell crawling tracks on immobilized recombinant VCAM-1 (1X, 1.54 μg/mL) **(D)**, ICAM-1 (1X, 1.14 μg/mL) **(E)**, and combined VCAM-1 (1X) / ICAM-1 (1X) **(F)** under physiological flow conditions are depicted for one representative experiment per group. For each track, the site of arrest was set to the center point of the respective diagram. End points of tracks are indicated by dots. Flow direction is illustrated by an arrow (yellow).

**Figure S5.** Percentage of human CD4^+^ Th1* adhesion inhibited by titrated bNTZ **(A,C)** and mNTZ **(B,D)** to immobilized recombinant VCAM-1 (1X) **(A,B)**, and combined VCAM-1 (1X) / ICAM-1 (1X) when β2-integrins are blocked (1 μg/mL anti-β2 antibody) **(C,D)** under flow condition. Red dotted line shows 95% limit of T cell adhesion inhibition. **(A-D)** Each figure shows the mean ± SEM of 3 experiments.
