# Supplementary material for: High levels of endothelial ICAM-1 prohibit natalizumab mediated abrogation of CD4+ T cell arrest on the inflamed BBB under flow in vitro
Source: J Neuroinflammation. 2023 May 23;20:123. doi: 10.1186/s12974-023-02797-8 (PMC10204262; doi:10.1186/s12974-023-02797-8)
Supplement: Supplementary file 7 — Additional file 7: Table S1. Antibody list for fluorescence-activated cell sorting of different CD4+ Th subsets. Table S2. Antibody list for flowcytometry analysis of adhesion molecules surface expression of BLEC, HBMEC and EECM-BMEC-like cells. Table S3. Antibody list for flowcytometry analysis of integrins surface expression of different CD4+ Th subsets. Table S4. Antibody list for immunofluorescence staining of adhesion molecules surface expression of BLEC, HBMEC and EECM-BMEC-like cells. [file 12974_2023_2797_MOESM7_ESM.docx]

**Table S1.** Antibody list for fluorescence-activated cell sorting of different CD4^+^ Th subsets

| **Target** | **Fluorophore** | **Clone** | **Provider** | **Cat. Number** | **Isotype** |
| --- | --- | --- | --- | --- | --- |
| **anti-human CD4** | PE-Tr | S3.5 | ThermoFischer Scientific | MHCD0417 | m-IgG2a, 𝜅 |
| **anti-human CD45RA** | Qd655 | MEM-56 | ThermoFischer Scientific | Q10069 | m-IgG2b, 𝜅 |
| **anti-human CD183 (CXCR3)** | AF647 | G025H7 | Biolegend | 353711 | m-IgG1, 𝜅 |
| **anti-human CD194 (CCR4)** | PE-Cy7 | 1G1 | BD Biosciences | 557864 | m-IgG1, 𝜅 |
| **anti-human CD196 (CCR6)** | PE | 11A9 | BD Biosciences | 559562 | m-IgG1, 𝜅 |
| **anti-human CD197 (CCR7)** | BV421 | G043H7 | Biolegend | 353208 | m-IgG2a, 𝜅 |
| **anti-human CD8** | PE-Cy5 | B9.11 | Beckman Coulter | A07758 | m-IgG1, 𝜅 |
| **anti-human CD14** | PE-Cy5 | RMO52 | Beckman Coulter | A07765 | m-IgG2a, 𝜅 |
| **anti-human CD19** | PE-Cy5 | HIB19 | ThermoFischer Scientific | 15-0199-42 | m-IgG1, 𝜅 |
| **anti-human CD25** | PE-Cy5 | B1.49.9 | Beckman Coulter | IM2646 | m-IgG2a, 𝜅 |
| **anti-human CD56** | PE-Cy5 | N901 | Beckman Coulter | A07789 | m-IgG1, 𝜅 |

**Table S2.** Antibody list for flowcytometry analysis of adhesion molecules surface expression of BLEC, HBMEC and EECM-BMEC-like cells

| **Target** | **Fluorophore** | **Clone** | **Provider** | **Cat. Number** | **Isotype** |
| --- | --- | --- | --- | --- | --- |
| **anti-human CD54 (ICAM-1)** | BV421 | HA58 | BD Biosciences | 564077 | m-IgG1, 𝜅 |
| **anti-human CD106 (VCAM-1)** | FITC | 51-10C9 | BD Biosciences | 551146 | m-IgG1, 𝜅 |

| **Isotype controls** | **Fluorophore** | **Clone** | **Provider** | **Cat. Number** |
| --- | --- | --- | --- | --- |
| **m-IgG1, 𝜅** | BV421 | X40 | BD Biosciences | 562438 |
| **m-IgG1, 𝜅** | FITC | MOPC-21 | BD Biosciences | 400108 |

**Table S3.** Antibody list for flowcytometry analysis of integrins surface expression of different CD4^+^ Th subsets

| **Target** | **Fluorophore** | **Clone** | **Provider** | **Cat. Number** | **Isotype** |
| --- | --- | --- | --- | --- | --- |
| **anti-human CD29**  **(**β**1-integrin)** | APC | MAR4 | BD Biosciences | 559883 | m-IgG1, 𝜅 |
| **anti-human CD49d**  **(**α**4-integrin)** | APC-Cy7 | 9F10 | Biolegend | 304328 | m-IgG1, 𝜅 |
| **anti-human** β**7-integrin** | BV650 | FIB504 | BD Biosciences | 564284 | rt-IgG2a, 𝜅 |

| **Isotype controls** | **Fluorophore** | **Clone** | **Provider** | **Cat. Number** |
| --- | --- | --- | --- | --- |
| **m-IgG1, 𝜅** | APC | MOPC-21 | BD Biosciences | 555751 |
| **m-IgG1, 𝜅** | APC-Cy7 | MOPC-21 | BD Biosciences | 400150 |
| **rt-IgG2a, 𝜅** | BV650 | R35-95 | BD Biosciences | 563144 |

**Table S4.** Antibody list for immunofluorescence staining of adhesion molecules surface expression of BLEC, HBMEC and EECM-BMEC-like cells

| **Target** | **Clone** | **Provider** | **Cat. Number** | **Isotype** | **Secondary Antibody** |
| --- | --- | --- | --- | --- | --- |
| **anti-human CD54 (ICAM-1)** | HA58 | Biolegend | 353102 | m-IgG1, 𝜅 | Cy3 AffiniPure F(ab')2 Fragment Goat Anti-Mouse IgG or AF488 Donkey Anti-Mouse Affine Pure IgG (H+L) |
| **anti-human CD 106 (VCAM-1)** | 51-10C9 | R&D systems | 555645 | m-IgG1, 𝜅 | Cy3 AffiniPure F(ab')2 Fragment Goat Anti-Mouse IgG or AF488 Donkey Anti-Mouse Affine Pure IgG (H+L) |
| **ZO-1** | polyclonal | Invitrogen | 40-2200 | N/A | Cy3 AffiniPure Donkey Anti-Rabbit IgG (H+L) |
